# Chloroplast Genome Sequence of Pigeonpea (*Cajanus cajan* (L.) Millspaugh) and *Cajanus scarabaeoides* (L.) Thouars: Genome Organization and Comparison with Other Legumes

**DOI:** 10.3389/fpls.2016.01847

**Published:** 2016-12-09

**Authors:** Tanvi Kaila, Pavan K. Chaduvla, Swati Saxena, Kaushlendra Bahadur, Santosh J. Gahukar, Ashok Chaudhury, T. R. Sharma, N. K. Singh, Kishor Gaikwad

**Affiliations:** ^1^ICAR-National Research Centre on Plant BiotechnologyNew Delhi, India; ^2^Department of Bio & Nanotechnology, Guru Jambheshwar University of Science & TechnologyHisar, India; ^3^Biotechnology Department, Biotechnology Centre, Dr. Panjabrao Deshmukh Krishi VidyapeethAkola, India

**Keywords:** *Cajanus cajan*, *Cajanus scarabaeoides*, chloroplast genome, Roche 454 sequencing, RNA editing

## Abstract

Pigeonpea (*Cajanus cajan* (L.) Millspaugh), a diploid (2n = 22) legume crop with a genome size of 852 Mbp, serves as an important source of human dietary protein especially in South East Asian and African regions. In this study, the draft chloroplast genomes of *Cajanus cajan* and *Cajanus scarabaeoides* (L.) Thouars were generated. *Cajanus scarabaeoides* is an important species of the *Cajanus* gene pool and has also been used for developing promising CMS system by different groups. A male sterile genotype harboring the *C. scarabaeoides* cytoplasm was used for sequencing the plastid genome. The cp genome of *C. cajan* is 152,242bp long, having a quadripartite structure with LSC of 83,455 bp and SSC of 17,871 bp separated by IRs of 25,398 bp. Similarly, the cp genome of *C. scarabaeoides* is 152,201bp long, having a quadripartite structure in which IRs of 25,402 bp length separates 83,423 bp of LSC and 17,854 bp of SSC. The pigeonpea cp genome contains 116 unique genes, including 30 tRNA, 4 rRNA, 78 predicted protein coding genes and 5 pseudogenes. A 50 kb inversion was observed in the LSC region of pigeonpea cp genome, consistent with other legumes. Comparison of cp genome with other legumes revealed the contraction of IR boundaries due to the absence of *rps19* gene in the IR region. Chloroplast SSRs were mined and a total of 280 and 292 cpSSRs were identified in *C. scarabaeoides* and *C. cajan* respectively. RNA editing was observed at 37 sites in both *C. scarabaeoides* and *C. cajan*, with maximum occurrence in the *ndh* genes. The pigeonpea cp genome sequence would be beneficial in providing informative molecular markers which can be utilized for genetic diversity analysis and aid in understanding the plant systematics studies among major grain legumes.

## Introduction

Pigeonpea (*Cajanus cajan* (L.) *Millspaugh*) popularly known as arhar, tur and red gram, is an important food legume crop, predominantly cultivated in tropical and subtropical regions of the world. It is a diploid (2n = 22) plant with an estimated genome size of 852 Mbp (Singh et al., 2012) and belongs to subfamily Papilionoideae, and family Leguminosae (Sharma and Green, 1980)

In the recent past, genome sequencing of pigeonpea has been reported (Varshney et al., 2011; Singh et al., 2012) along with the, mitochondrial genome (Tuteja et al., 2013) but chloroplast genome sequencing has not been decoded so far. The first complete chloroplast (cp) genome sequences to be decoded were of tobacco and liverwort in 1986 (Ohyama et al., 1986; Shinozaki et al., 1986). Till date, the chloroplast genome sequences of a number of land plants and algae have been reported. Among the land plants, 888 complete chloroplast genomes have been sequenced till date^1^ and which includes 44 genomes belonging to the Leguminosae family are available including those of for example *Cicer arietinum* (Jansen et al., 2008), *Trifolium subterraneum* (Cai et al., 2008), *Phaseolus vulgaris* (Guo et al., 2007), *Lotus japonica* (Kato et al., 2000), *Glycine max* (Saski et al., 2005), *Medicago truncatula* (Young et al., 2011), and *Vigna radiata* (Tangphatsornruang et al., 2010). Sequencing of complete plastid genomes has been made easy by development of next generation sequencing technologies. The first attempt to use next generation sequencing technology (454 GS 20 system) for the sequencing of chloroplast genome was made by Moore et al. (2006). As the genetic features of the chloroplast genome are relatively simple, it has contributed to the study of molecular systematics and DNA barcoding (Dong et al., 2012). Uniparental inheritance, low level of recombination and lower substitution rates in comparison to nuclear genome, makes chloroplast genome sequence useful for phylogenetic analysis (Provan et al., 2001) and species identification (Li et al., 2015).

A typical plant chloroplast genome consists of single circular chromosome with a quadripartite structure, which includes two copies of an inverted repeat (IR) spanning 12–75 kb that separates the large and small single copy regions, LSC (80–90 kb) and SSC (16–27 kb). Expansion, contraction or loss of the IR and variation in length of intergenic spacers leads to variation in length of genomes but generally the size of chloroplast genome of photosynthetic organisms ranges between 115 and 165 kb (Palmer, 1991; Raubeson and Jansen, 2005). A typical angiosperm chloroplast contains 110–130 genes comprising of 4 rRNA, 30–31 tRNA, and 80–90 protein coding genes. The IR region comprises of a duplicated set of tRNA and rRNA genes while the single copy regions mostly consists of the genes encoding ribosomal proteins, RNA polymerase subunits, proteins associated with photosystems, as well as protein subunits for NADH dehydrogenase complex. The two IRs are inverted replicas of each other and hence the genes in the IR are present in two copies (Bock, 2007). Increased gene dosage and genome stabilization have been proposed as the reason for the presence of two copies of IR but absence of one copy of IR from some higher plant cp genomes have shown that it is dispensable for the plastome function (Palmer and Thompson, 1982).

Even though chloroplast genome structure seems to be highly conserved among plants, there are some differences in terms of gene synteny and copy number. For instance, gene duplications are reported for few tRNA genes, *ycf2, rpl23*, and *psbA* in some and loss of *accD, psaI, rpl23, rps16, ycf4*, and *infA* in others (Jansen et al., 2007; Magee et al., 2010). It is also reported that ndhF and ycf2 genes were lost repeatedly from a variety of angiosperms during the course of evolution (Shinozaki et al., 1986; Wolfe et al., 1992; Sato et al., 1999). Pseudogenes are also observed in various land plants like *ycf2* which is responsible for cell viability in rice and maize (Hiratsuka et al., 1989; Maier et al., 1995), *infA* gene (translation initiation factor) in tobacco, *Arabidopsis* and *Oenothera elata* and *rpl23* gene in spinach (Thomas et al., 1988). In contrast, cp genomes of the plants belonging to fabaceae family have been reported to undergo extensive rearrangements as compared to other angiosperms (Kato et al., 2000; Guo et al., 2007; Cai et al., 2008; Jansen et al., 2008) Complete loss of the inverted repeat (IR) which occurred rarely during evolution of angiosperm has been reported in pea (Palmer and Thompson, 1981).

The loss of one copy of IR has occurred in a large clade of papilionoid legumes which includes the tribes *Carmichaelieae, Cicereae, Galegeae, Hedysareae, Trifolieae*, and *Fabeae*. The monophyly of IR- lacking clade (IRLC) (Wojciechowski et al., 2000) has been confirmed with the help of phylogenetic analysis by using plastid genes *matK* (Wojciechowski et al., 2004), *rbcl* (Doyle et al., 1997), *trnL* intron (Pennington et al., 2001), and ITS regions of nuclear ribosomal DNA (Hu et al., 2002). The chloroplast genomes sequenced from IRLC includes: *Trifolium aureum; T. repens; T. grandiflorum* and *T. subterraneum* (clover); *C. arietinum* (chickpea); *M. truncatula* (barrel medic); *Pisum sativum* (pea); *Lathyrus sativus* (grass pea); *Lens culinaris* (lentil); *Glycyrrhiza glabra* (licorice); and *Vicia faba* (broad bean) (Saski et al., 2005; Cai et al., 2008; Jansen et al., 2008; Magee et al., 2010; Sabir et al., 2014). Also it is now believed that loss of IR made the chloroplast genome more prone to rearrangements, like a 50 kb inversion reported in mung bean (Palmer and Thompson, 1982), is present in most members of the papilionoideae subfamily which changes the gene order between *trnK* and *accD* genes in the LSC region (Palmer et al., 1988). Another inversion encompassing a 78 kb region in LSC was first reported in *Phaseolus* and *Vigna*, a member of subtribe phaseolinae and tribe phaseoleae (Bruneau et al., 1990; Guo et al., 2007; Tangphatsornruang et al., 2010) and a newly reported 36 kb inversion within the 50 kb inversion present in lupines and other genisotoids (Martin et al., 2014). There seems to be variation in the legumes for the presence of certain genes. Genes *infA* and *rpl22* are not encoded by legume chloroplasts (Doyle et al., 1995), rather it is reported that their nuclear copies are being directed toward the chloroplast (Gantt et al., 1991; Millen et al., 2001). The *accD* gene is also reported to be functionally transferred to the nucleus in *Trifolium* species (Magee et al., 2010). The loss of intron in the *clpP* and *rps12* genes has also been mapped to Leguminosae phylogeny (Doyle et al., 1995; Jansen et al., 2008).

Microsatellites or Simple sequence repeats (SSRs) are short DNA sequence stretches in which a motif of one to six bases is tandemly repeated (Schlötterer, 2000). Powell et al. (1995) reported likewise nuclear SSRs, chloroplast microsatellites also demonstrate significant polymorphism. Chloroplast SSRs demonstrate high level of intraspecific variation and thus are considered as potential markers in evolutionary, population and systematics studies in plants (Provan et al., 2001).

Of late, cp genome sequencing has acquired new dimensions. Recent methods like amplification of entire genome using rolling circle amplification (Dhingra and Folta, 2005), high throughput sequencing (Moore et al., 2006; Cronn et al., 2008; Yan et al., 2015) have been successful in achieving fast and cost effective chloroplast genome sequencing. Pigeonpea genomics is gathering speed and that requires availability of all types of genomics resources. The sequence of plastid genomes of pigeonpea will aid in effective utilization for genotyping. Here we report the use of Roche 454 FLX sequencing technology for obtaining draft chloroplast genome sequence of *Cajanus cajan* and *Cajanus scarabaeoides* for understanding the genome organization, editing changes and mining of SSR markers.

## Materials and methods

### Plant material and DNA isolation

Cytoplasmic male sterile pigeonpea AKPA1 (*C. scarabaeoides* cytoplasm) and its fertility restorer AKPR375 (*C. cajan* cytoplasm) were used in this study. Fresh leaves were harvested from seedlings and were kept in the dark for 48 h prior to chloroplast DNA isolation. Chloroplast DNA isolation was performed as per Kirti et al. (1993).

### Chloroplast genome sequencing, assembly and annotation

The plastid DNA (1 μg) was sheared by nebulization and purified to obtain the desired size range. Library preparation and sequencing by Roche 454 GS FLX platform was carried out as per manufacturer's instructions. Two biological replicates were later pooled for data analysis.

Pyrosequencing was performed on a Genome Sequencer FLX system using Titanium Chemistry (Roche, 454). The per base quality of the raw reads (496,972, 498,603) was assessed by FastQC V0.11.4^2^. Quality filtering was done using PRINSEQ lite V0.20.4 (Schmieder and Edwards, 2011; phred Q ≥ 20, Length ≥ 50). Quality filtered reads were *denovo* assembled using Newbler (GS *de novo* Assembler) v2.6 programme with default parameters.

Contigs larger than 200 bp were extracted to construct consensus using *G. max* chloroplast genome. Contigs were aligned to *G. max* cp genome sequence by BLASTN (https://www.nih.gov/). Contigs with >80% matches were ordered against the reference. Gap between adjacent contigs was initially filled with “N” to construct consensus cp genome. The gaps in the genome were filled by alignment of filtered reads using CLC Genomics Workbench 7.5.1 (CLC Bio, Arhus, Denmark) with following parameters: Length fraction = 0.5, Similarity = 0.9 to the end and gap filling extended read-contig regions were merged where 10 bp or more bp overlapping till a single large fragment was obtained.

Genome annotation was carried out with DOGMA (Dual Organellar Genome Annotator; Wyman et al., 2004) to identify coding sequences (cds), rRNAs, and tRNAs using the plastid genetic code and BLAST homology searches. To verify the exact gene and exon boundaries, we compared Pigeonpea annotations with those of *G. max* and manually corrected the start and stop codons. The presence of tRNA genes were also confirmed by online tRNAscan-SE 1.21 search serve (Lowe and Eddy, 1997).

The entire cp sequences of *C. scarabaeoides* and *C. cajan* genotypes, along with gene annotations were submitted to GenBank (accession number: KU729878 for *C. scarabaeoides* and KU729879 for *C. cajan*).

### PCR amplification

To confirm the junctions between LSC and IR; SSC and IR, PCR amplification was carried out in a total reaction volume of 20 μl containing 30 ng of DNA template, 1 × buffer, 0.2 mM dNTPs, 2.5 mM MgCl_2_, 1U DNA Polymerase and 0.5 μM each of forward and reverse primers. Primer pair—(i) LI_F1: TCCCTCGACACCAGAAGATA, LI_R1: CCGGATCTAAATGTTGGCTA, (ii) LI2_F2: GTCGGACAAGTGGGAAATGT, LI2_R2: CCGAGCTAACCTTGGTATGG were used to amplify the junction between LSC and IR. And the primer pair—(i) SI_F1: GTTGGTTTAAATAGCCCCG; SI_R1:CCATCTGTTAACCATTTTTGGGG, (ii) SI_F2:TGTGATTATTGCCGAAGAACTG,SI_R2:CGTTCTCAACCCATGACCAA were used to amplify the junction between SSC and IR. Amplification was performed in Techne PCR: 94°C for 3 min followed by 40 cycles of 94°C for 30 s, 52°C for 30 s, 72°C for 1 min and a final extension step at 72°C for 10 min. Amplified products were separated on a 1.2% agarose gel.

### Genome analysis

Full alignments of legume cp genomes were performed using mVISTA program (Frazer et al., 2004) in Shuffle-LAGAN mode. Selected legume cp genomes were retrieved from NCBI: *G*. *max* (NC_7942), *P. vulgaris* (NC_9259), *Cicer areitinum* (NC_11163), *V. radiata* (NC_13843) and used as a reference.

The comparison of gene order between the chloroplast genomes of *C. cajan, C. scarabaeoides, Arabidopsis thaliana* (NC_000932), *G. max* (NC_7942), *P. vulgaris* (NC_9259), *C. areitinum* (NC_11163), *V. radiata* (NC_13843), and *M. truncatula* (NC_003119) was performed with MAUVE (Darling et al., 2004). Codon usage was calculated for all exons of protein-coding genes with CodonW 1.4.4. Base composition was calculated by DNA/RNA base composition calculator^3^.

### RNA editing analysis

Predictive RNA Editor for Plants (PREP) suite^4^ was used to predict RNA editing sites (Mower, 2009). For the analysis, the cut-off value was set at 0.8. The PREP-cp program consists of 35 reference genes for predicting RNA editing sites in the chloroplast genomes. The editing sites were validated by mapping the transcriptome data (unpublished data) onto the DNA sequences from the chloroplast in CLC Genomics Workbench 7.5.1 (CLC Bio, Arhus, Denmark). The sites having more than 5X coverage (C–U) were considered as true editing changes.

### SSR analysis

Chloroplast microsatellites (cpSSRs) were identified in high quality sequence of *C. scarabaeoides* and *C. cajan* by using MISA^5^ perl script. The identified cpSSRs included mononucleotide repeats ≥ 8 bases, dinucleotides ≥ 10 bases (five repeats) and trinucleotides and tetranucleotides ≥ 12 bases (four and three repeats respectively), pentanucleotide ≥ 15 bases (3 repeats) and hexanucleotides ≥ 18 bases (3 repeats).

## Results and discussion

### Chloroplast genome assembly

Roche-454 Sequencing of *C. scarabaeoides* and *C. cajan* chloroplast genomes from purified DNA generated about 496,972 and 498,603 reads respectively. Filtered reads (496,228 and 497,800) were used for *de novo* assembly using Newbler (v.2.6 454 Life Science). A total of 13,732 (N50, 900 bp) and 13,002 (N50, 889 bp) contigs from *C. scarabaeoides* and *C. cajan* were respectively obtained with size ranging from 200 to 79,709 bases. They were then organized by using *G. max* chloroplast as reference. The contigs with >80% matches were used to build a draft consensus. Finally, to fill gaps in the consensus, filtered reads were aligned to draft consensus and the sequence of the read-contig in the direction of the gap were compared. If there was an overlap of 10 bp or more, the two contigs were joined together. Using this strategy, we achieved a minimum coverage of 99.96% of the cp genome for the *C. scarabaeoides* and *C. cajan* chloroplast genome. The size of cp genomes of *C. scarabaeoides* and *C. cajan* was found to be 152,201 bp and 152,242 bp. Finally, the four junctions between IRs and LSC/SSC were confirmed and validated by PCR amplification.

### Genome content and organization of the pigeonpea plastid genome

**Figure 1:**
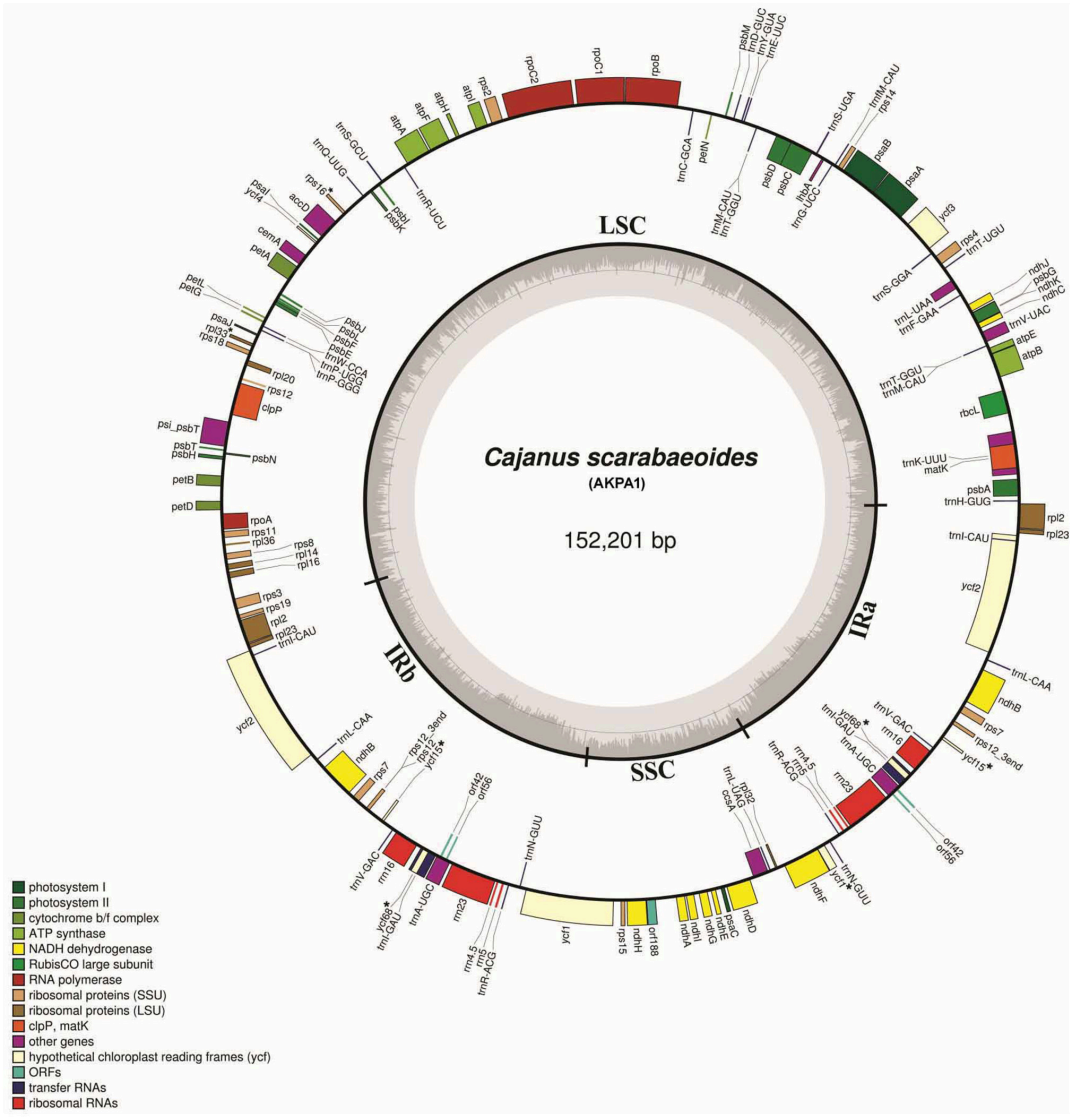
**Map of *C. scarabaeoides* plastid genome**. Genes shown on the outside of the map are transcribed clockwise while the genes that are shown on the inside are transcribed counterclockwise. The innermost darker gray corresponds to GC content, whereas the lighter gray corresponds to AT content. Different genes are color coded. IR, inverted repeat; LSC, large single copy region; SSC, small single copy region. Pseudogenes are marked with “^*^.”

**Figure 2:**
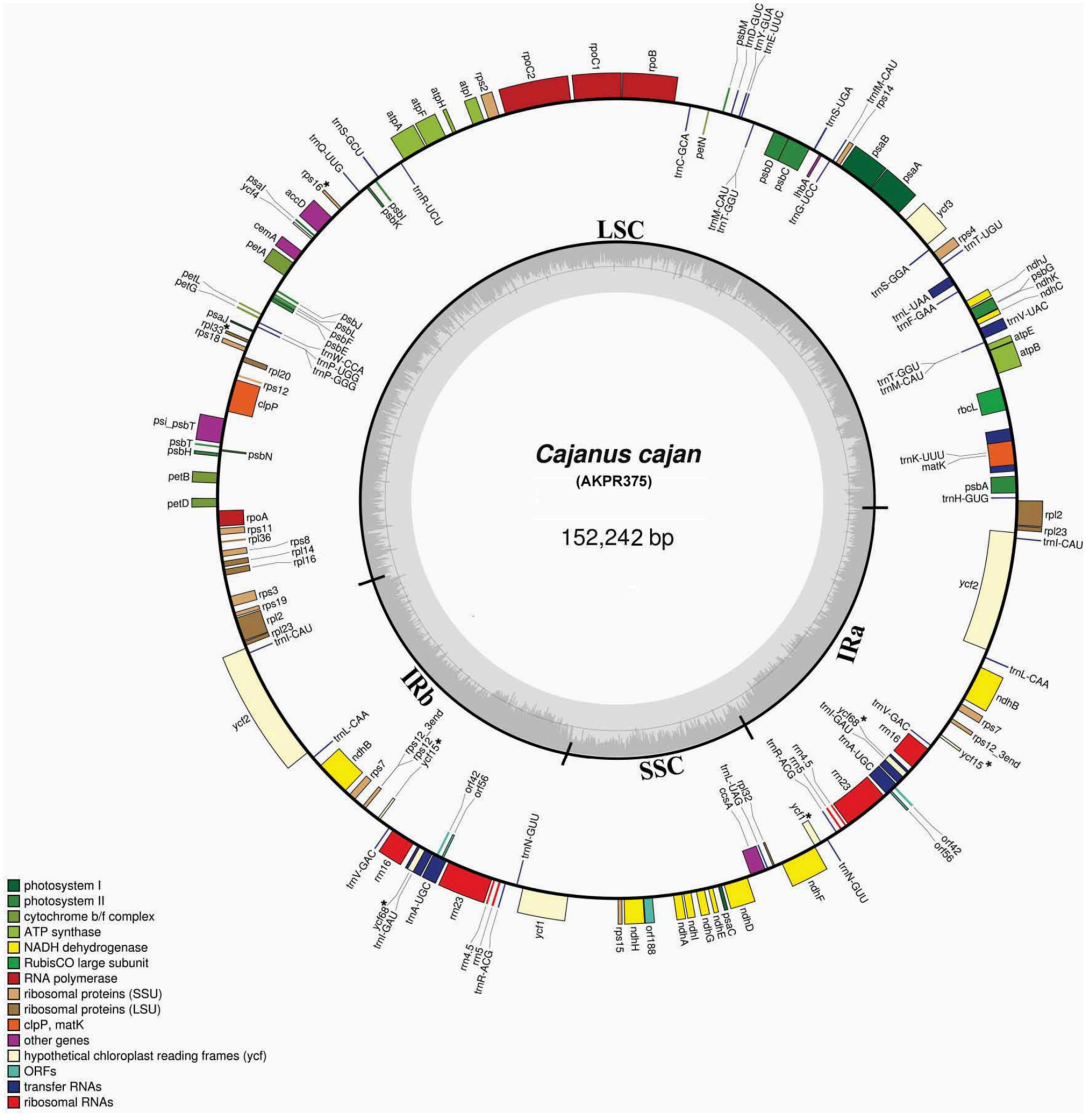
**Map of *C. cajan* plastid genome**. Genes shown on the outside of the map are transcribed clockwise while the genes that are shown on the inside are transcribed counterclockwise. The innermost darker gray corresponds to GC content, whereas the lighter gray corresponds to AT content. Different genes are color coded. IR, inverted repeat; LSC, large single copy region; SSC, small single copy region. Pseudogenes are marked with “^*^.”

The cp genomes of *C. scarabaeoides* and *C. cajan* are 152,201 bp and 152,242 bp in length respectively. It consists of a quadripartite structure with IRs of 25,402 bp separating 83,423 bp of LSC and 17,854 bp of SSC in *C. scarabaeoides*, while 25,398 bp of IR separates 83,455 bp of LSC and 17,871 bp of SSC in *C. cajan* (Figures 1, 2). The cp genome of *C. scarabaeoides* and *C. cajan* differs slightly from *G. max* (152,218 bp) and other legumes (*V. radiata*-151,271 bp; *P. vulgaris*-150,285 bp; *C. arietinum*-125,319 bp) in terms of size, (Supplementary Table S1).

Both plastid genomes contain 116 unique genes, which include 30 tRNA, 4 rRNA, 78 predicted protein coding genes and 5 peudogenes. The LSC region consists of 58 protein coding genes and SSC region consists of 13 protein coding genes in both genotypes. The tRNA coding genes represents 20 amino acids in both genotypes and are distributed throughout the genome, with one tRNA coding gene present in the SSC region, 22 in the LSC region and 7 in the IR region of both. For the 61 possible codons (excluding stop codon), 28 tRNAs exist in the cp genome of both genotypes. The *trnT-GGU* and *trnM-CAU* genes are duplicated in the LSC region of both cp genomes. Similar tRNA genes duplications have also been reported in the cp genome of *Actinidia*, black pine and green algae (Tsudzuki et al., 1994; Wakasugi et al., 1997; Yao et al., 2015). The IR region consists of 7 tRNA coding genes, 4 rRNA coding genes, 8 protein coding genes (*rpl2, rpl23, ycf2, ndhB, rps7, rps12, orf42*, and *orf56*) and 2 pseudogenes (*ycf15* and *ycf68*) in both *C. scarabaeoides* and *C. cajan* (Table 1), thus these genes seem to be generally duplicated in the IR regions. Therefore, in total 138 genes are present in the cp genome of pigeonpea (Figures 1, 2). Trans-splicing is observed in *rps12* gene with 5′ end exon present in the LSC region and the 3′ end exon duplicated and present in the IR region.

**Table 1:** **List of genes present in the cp genome of *C. scarabaeoides* and *C. cajan***.

**Category**	**Gene Name**
Photosystem I	*psaA,B,C,I,J,Ycf3^a^,ycf4*
Photosystem II	*psbA,B,C,D,E,F,H,I,J,K,L,M,N,T,Z/lhbA*
Cytochrome b6/f	*petA,B,D,G,L,N*
ATP Synthase	*atpA,B,E,F^b^,H,I*
Rubisco	*rbcL*
NADH Oxidoreductase	*ndhA,B^b^^,^^c^,C,D,E,F,G,H,I,J,K*
Large subunit ribosomal proteins	*rpl2^b^^,^^c^,14,16,20,23^c^,32,33^e^,36*
Small subunit ribosomal proteins	*rps2,3,4,7^c^,8,11,12^c^^,^^d^,14,15,16^e^,18,19*
RNAP	*rpoA,rpoB,C1^b^,C2*,
Other Proteins	*accD, ccsA,matK,cemA,clpP^a^*,
Proteins of unknown Function	*ycf1^c^^,^^e^,ycf2^b^^,^^c^, ycf15^c^^,^^e^,ycf68^c^^,^^e^,orf42^c^,orf56^c^,orf188*
Ribosomal RNAs	*rrn23^c^,16^c^,5^c^,4.5^c^*
Transfer RNAs	*trnH(GUG), K(UUU)^b^,M(CAU),T(GGU),V(UAC)^b^,F(GAA),L(UAA)^b^,T(UGU),S(GGA),fM(CAU), G(UCC),S(UGA),E(UUC),Y(GUA),D(GUC),C(GCA),R(UCU),S(GCU),Q(UUG), W(CCA), P(UGG), P(GGG), I(CAU)^c^, L(CAA)^c^, V(GAC)^c^, I(GAU)^b^^,^^c^, A(UGC)^b^^,^^c^, R(ACG)^c^, N(GUU)^c^,L(UAG)*

a *Gene containing two introns*.

b *Gene containing a single intron*.

c *Two gene copies in the IRs*.

d *Gene divided into two independent transcription units*.

e *Pseudogenes*.

The average AT content of the cp genome is 66% for both genotypes (Table 2), which is found to be in similar range, reported for the legumes including *G. max* (64.63%) and *C. arietinum* (66.1%). Individually the AT content of the LSC and SSC regions is 68% and 72% in both *C. scarabaeoides* and *C. cajan*. The AT content of IR region is 58% in both and is consistent with findings for other cp genomes. The low AT content of the IR region may be due to the reduced presence of AT nucleotides in the four rRNA genes (*rrn16, rrn23, rrn5*, and *rrn4.5*) present in IR region. The increased sequence complexity of the IR regions may help in the stabilization of the genome as it has been reported in the past that the legume plastids which have lost one copy of IR are more prone to rearrangements as compared to those genomes which have retained the IR copy (Palmer and Thompson, 1982).

**Table 2:** **Features of the chloroplast genome of *C. scarabaeoides* (Cs) and *C. cajan* (Cc)**.

	**T/U%**		**C%**		**A%**		**G%**		**Length (bp)**		**AT%**	
	**Cs**	**Cc**	**Cs**	**Cc**	**Cs**	**Cc**	**Cs**	**Cc**	**Cs**	**Cc**	**Cs**	**Cc**
Genome	33	33	17	17	33	33	18	18	152,201	152,242	66	66
LSC	34	34	16	16	34	34	17	17	83,423	83,455	68	68
SSC	36	36	13	13	36	36	15	15	17,854	17,871	72	72
IR	29	29	20	20	29	29	22	22	25,402	25,398	58	58
Prt.Coding genes	32	32	17	17	31	31	19	19	79,052	75,031	63	63
tRNA	26	25	23	23	23	23	28	29	2888	2888	48	48
rRNA	19	19	23	23	26	26	32	32	9054	9054	45	45
First position	32.1	32.1	17.5	17.5	31.8	31.8	18.3	18.3	50,747	50,747	64	64
Second position	33.02	33.02	17.23	17.23	32.77	32.77	16.96	16.96	50,747	50,747	65.79	65.79
Third position	32.3	32.3	16.6	16.6	32.9	32.9	18.1	18.1	50,747	50,747	64.5	64.5

Protein coding regions account for 49.2% of the whole genome while tRNA and rRNA accounts for 1.9% and 5.9% respectively in *C. cajan*, whereas in *C. scarabaeoides*, protein coding region accounts for 51.9% while 1.9% and 5.9% are accounted by tRNA and rRNA regions respectively. The remaining region consists of non-coding sequences which include intergenic regions, introns and pseudogenes.

In the cp genome of *C. scarabaeoides*, a total of 79,052 nt and 26,350 codons represent the coding capacity of 78 protein coding genes. Among these, leucine (2898 codons, 10.99% of the total) represents the most abundant amino acid whereas cysteine (354 codons, 1.34% of the total) represents the least abundant amino acid. Similarly, in the *C. cajan*, 78 protein coding genes are represented by 75,031 nt and 25,010 codons. Here too leucine (2264 codons, 9.05%) is the most abundant amino acid and cysteine (416 codons, 1.66%) is the least abundant amino acid (Supplementary Tables S2, S3). Leucine and cysteine are reported as the most and least abundant amino acids respectively in other cp genomes also (Chen et al., 2015; Curci et al., 2015; Redwan et al., 2015). It has been suggested in previous studies that there is a significant relationship between codon usage bias and gene expression level (Iannacone et al., 1997; Rouwendal et al., 1997), therefore it implies that there is a strong natural selection pressure on highly expressed genes to optimize their translation efficiency by using major codons (Bulmer, 1988). The codon usage is biased toward the high representation of A and T at the third codon position (Table 2). The biasness for A and T nucleotide at third codon position is also shown by RSCU analysis for instance, for valine the codon ending with A and T are 36.5% whereas those ending with G and C are 14.75 and 12.25% respectively. Such biasness for high representation of A and T at third codon position is also observed in other land plant plastid genomes (Yang et al., 2014; Yao et al., 2015). It may be due to the compositional bias toward AT rich content (Morton, 2003; Williams et al., 2015). As all cp genomes have high AT content, AT biased mutational pressure and its prokaryotic origins are believed to be the factors responsible for codon usage bias.

There are 12 intron containing genes in both the genotypes. Among these, 10 genes (5 protein coding genes and 5 tRNA genes) have a single intron and 2 genes (*ycf3* and *clpP*) have two introns each. *Cicer, Medicago, Trifolium, P. sativum*, and *L. sativus* has lost the *clpP* introns and this loss provides support for the monophyly of IRLC (Jansen et al., 2008). On the other hand, *Acacia liguata*, a member of Mimosoideae subfamily of legumes retains both the introns of clpP. The intron containing genes are distributed throughout the genome with 7 genes present in the LSC region and 5 genes present in IR region of both the genotypes (Supplementary Tables S4, S5). Among the intron containing genes, *trnK-UUU* has the largest intron in both the plastids (2593 bp in *C. scarabaeoides* and 2594 bp in *C. cajan*) and likewise this intron also contains *matK* gene, which is consistent with other legume plastid genomes (Saski et al., 2005; Tangphatsornruang et al., 2010). Koch et al. (1981) demonstrated for first time the presence of intron in cp *trna* genes, *trnI*, and *trnA*. Some recent studies have suggested that, introns play an important role in the regulation of gene expression and therefore improve exogenous gene expression, resulting in the enhanced plastome efficiency (Xu et al., 2003).

It was observed that *rpl22* and *infA* genes are absent from the plastid genome of both genotypes. The absence of *rpl22* gene is also observed in *G. max* (Saski et al., 2005), *T. subterraneum* (Cai et al., 2008), and *Lotus japonicus* (Kato et al., 2000). Molecular analysis suggested the transfer of *rpl22* gene to nucleus from the cp genome, as a functional copy of this gene has been found from the nuclear genome of *P. sativum* (Gantt et al., 1991). Also a functional copy of *rpl22* gene was verified in the nucleus of lupine species (Martin et al., 2014). The gene *infA* has been lost from cp genome to nucleus in the course of angiosperm evolution in almost all rosids (Millen et al., 2001). A pseudogene *rps16* is present in the plastid genome of both *C. scarabaeoides* and *C. cajan*, whereas it has been lost from the genome of *C. arietinum* (Jansen et al., 2008), *M. truncatula* (Young et al., 2011) and is present as a non-functional copy in *V. radiata* (Tangphatsornruang et al., 2010). The loss of *rps16* has occurred multiple times from the legumes (Doyle et al., 1995). Gene substitution has been identified as the mechanism for loss of *rps16* gene from cp genomes of *Populus* and *Medicago* (Ueda et al., 2008). The dual targeting of mt. ribosomal protein S16 (encoded by nuclear gene) to mitochondria as well as to chloroplast compensates for the loss of cp *rps16* gene. Another gene, *rpl33* observed to be present in *C. scarabaeoides, C. cajan, Vigna*, and *Phaseolus* (Guo et al., 2007) is also a pseudogene as it contains a premature stop codon within the coding region.

Among the five completely sequenced legume plastid genomes, three genomes (*Cicer, Glycine*, and *Medicago*) lack the *ycf4 gene* whereas it is present in both *C. scarabaeoides* and *C. cajan*. Magee et al. (2010) identified a 1.5 kb long region having dramatically high rate of evolution coinciding with *ycf4* gene. It has been found that *ycf4* has evolved much faster in most legumes than in other angiosperms. It is reported to be lost from the cp genome of *Lathyrus odoratus* (Magee et al., 2010) and either absent or present as a pseudogene in *P. sativum* (Nagano et al., 1991; Smith et al., 1991). It has been established by slot-blot hybridization experiments that ycf4 may have been lost independently multiple times in different lineages of legumes (Doyle et al., 1995). Magee et al. (2010) also reported a very interesting finding that ycf4 gene which was reported absent from the cp genome of *G. max, T. subterraneum, Cicer arientinum* and *M. truncatula* was present in all the cp genomes but as the gene is so divergent, DOGMA (Wyman et al., 2004) was not able to annotate them.

The two pseudogenes *ycf15* and *ycf68* present in *C. scarabaeoides* and *C. cajan*, seem to contain premature stop codons, similar to that observed in *V. radiata* and *P. vulgaris.* In Artichoke, *ycf68* is reported to be a pseudogene (Curci et al., 2015), while both *ycf15* and 68 are reported as pseudogenes in sweet potato (Yan et al., 2015). The *accD* gene which was reported to be relocated to nucleus in *Trifolium* species (Cai et al., 2008) is present in both *C. scarabaeoides* and *C. cajan*, and all the other sequenced legumes (Guo et al., 2007; Jansen et al., 2008). It has been reported that *accD* shows considerable length variation among the legumes that retains it. The increased rate of sequence evolution and localized hypermutation has led to the phenomenon of gene loss or relocation to nucleus in legumes (Magee et al., 2010). Among the angiosperms, legumes are more prone to rearrangements and gene losses (Palmer et al., 1988). Mostly the genes coding for ribosomal proteins have been lost during the evolution from the plastid genome. There are no reports for the loss of genes related to electron transport chain, atp synthesis or those associated with photosystem I and II (Jansen et al., 2007).

### Gene order

**Figure 3:**
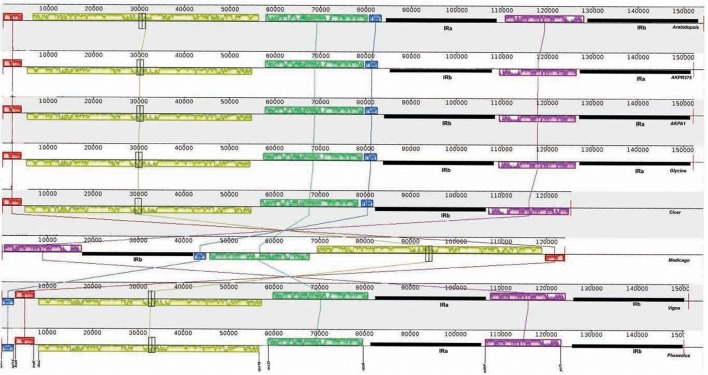
**Gene order comparison of legume cp genomes, with Arabidopsis cp genome as reference, using MAUVE software**. The boxes above the line represent the gene sequence in clockwise direction and the boxes below the line represent gene sequences in opposite orientation. The gene names at the bottom indicate the genes located at the boundaries of the boxes in cp genome of pigeonpea. AKPA1- *C. scarabaeoides*, AKPR375*- C. cajan.*

The cp genomes of *C. cajan* and *C. scarabaeoides* were aligned with the cp genomes of previously reported legumes by including *Arabidopsis* cp genome as reference with help of Mauve software (Darling et al., 2004; Figure 3). All the legume cp genomes generally shared the same gene order but the major difference among them was absence of IRb region in *Cicer* and *Medicago*. The cp genome of *Cicer* has lost one copy of the IR, a feature also shared by *Medicago*. Lavin et al. (1990) reported the loss of one copy of inverted repeat in six legume tribes including Galegeae, Hedysareae, Carmichaelieae, Vicieae, Cicereae, and Trifolieae. All these legume tribes form a new clade called IRLC (inverted-repeat-lacking clade; Palmer et al., 1987; Cronk et al., 2006). The cp genomes possessing the inverted repeat have a very conserved and stable genomic structure while the genomes which have lost one copy of inverted repeat have undergone extensive genomic rearrangements (e.g., *Vicia, Trifolium, Pisum*; Palmer and Thompson, 1982; Doyle et al., 1995).

All the legume genomes have a common 50-kb inversion as compared to *Arabidopsis* cp genome. This inversion spans the region between *rbc*l and *rps16* in the LSC region. This inversion was described for the first time in *P. sativum, V. faba*, and *V. radiata* (Palmer and Thompson, 1982) and is confined to Papilionoideae subfamily of leguminosae (Doyle et al., 1996).

Another inversion of 78-kb is present in cp genome of *V. radiata* and *P. vulgaris* but absent from other legume cp genomes, was originally reported in subtribe phaseolinae (*Vigna* and *Phaseolus*). The inversion spans the region between *trnH-GUG/rpl14* and *rps19/rps8*. This 78-kb inversion may have resulted due to expansion and subsequent contraction of the inverted repeats (Bruneau et al., 1990).

The cp genome of both pigeonpea genotypes displays one more inversion between the LSC and IRs which is common with *G. max*. This may be the result of flip-flop intramolecular recombination occurring in the plastome (Palmer, 1983). The rearrangements such as inversions in the chloroplast genome of land plants are rare and they have proven to be useful markers for phylogenetic analysis (Jansen and Palmer, 1987; Doyle et al., 1992; Raubeson and Jansen, 1992) in a number of groups such as legumes (Bruneau et al., 1990). Therefore, these rearrangements are indicative of the diversity observed in the cpDNA organization of legume plants.

### Comparison with other legume genomes

**Figure 4:**
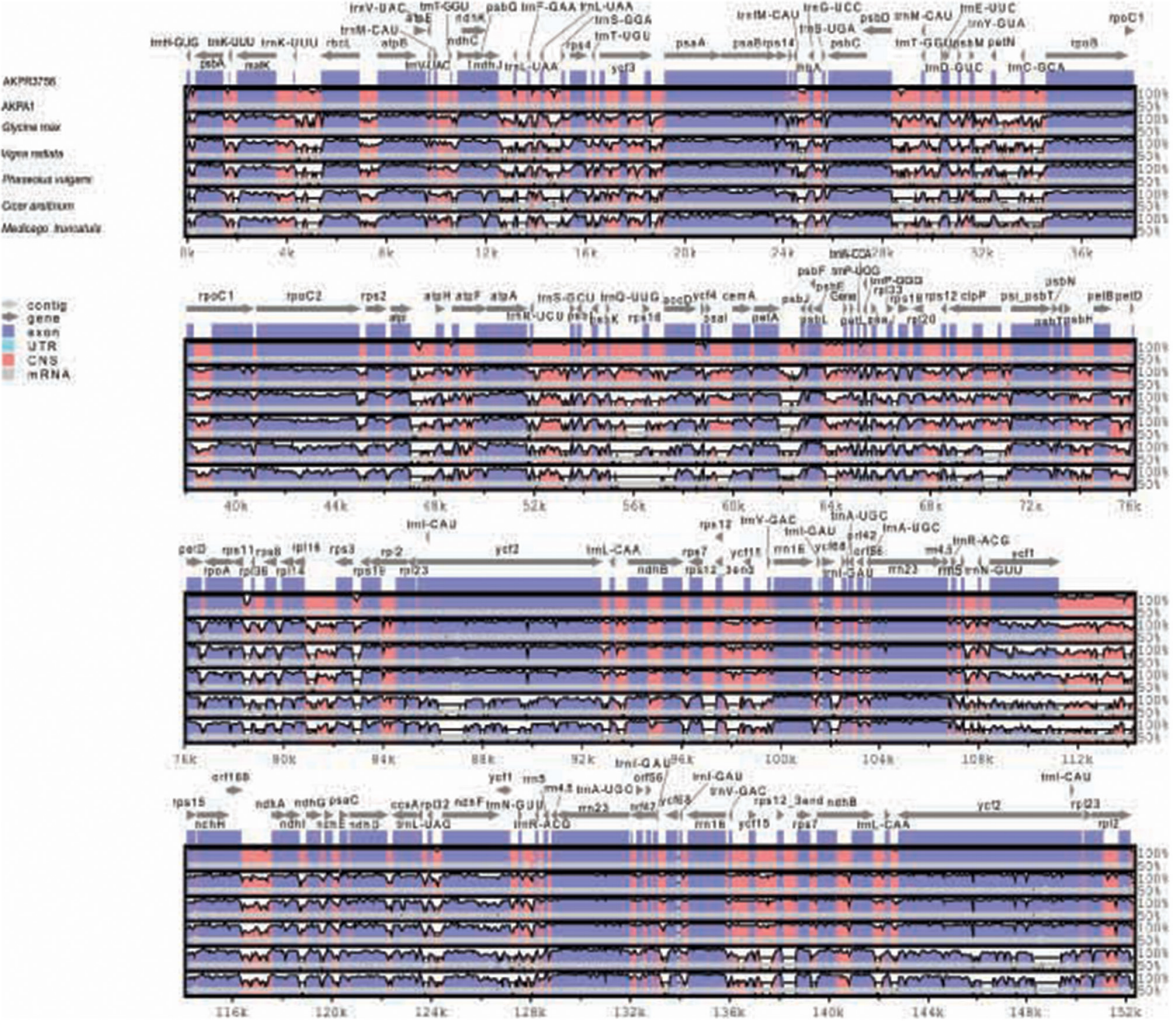
**Sequence alignment of legume cp genomes, with *C. cajan* cp genome set as a reference using mVista**. Position and transcriptional direction of each gene is indicated by gray arrows. Intergenic and genic regions are indicated by red and blue areas respectively. Sequence identity between the cp genomes is shown on y-axis as a percentage between 50 and 100%. AKPA1- *C. scarabaeoides*, AKPR375*- C. cajan.*

The sequence identity of *C. scarabaeoides* and *C. cajan* cp genome was plotted using mVISTA (Figure 4). The coding regions were found to be more conserved than the non-coding regions, as also reported for other cp genomes. The IR regions were found to be more conserved than the single copy regions probably due to the phenomenon of copy correction between IR sequences by gene conversion (Khakhlova and Bock, 2006). Another explanation for the conservation of IR is the presence of conserved rRNA genes in the IR region. The coding regions showing high degree of variation are *accD, cemA, petA, psbT*, and *clpP* as also reported for other cp genomes (Yang et al., 2014; Yao et al., 2015). The intergenic region between *trnC-GCA–psbD, petD-rps3, psbK-accD, petA-psbT trnK-UUU- rbcL*, and *ndhJ–ycf3* show high sequence divergence among the legumes aligned.

### IR boundaries

**Figure 5:**
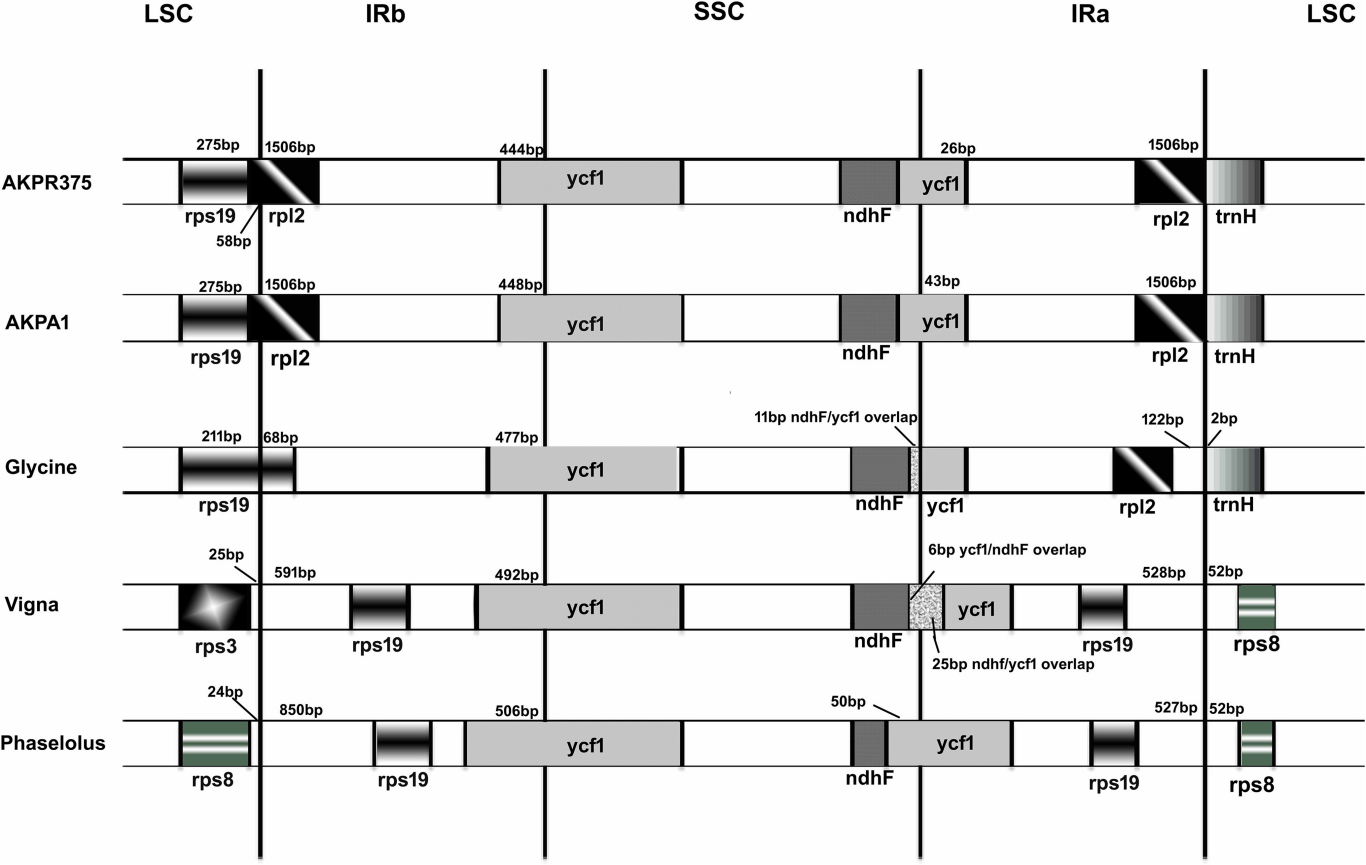
**Comparison of the border positions of LSC, SSC, and IR regions among the legume genomes**. Genes are denoted by boxes and the gaps between the genes and the boundaries are indicated by number of bases unless the gene coincides with the boundary. Extensions of the genes are also indicated above the boxes. AKPA1- *C. scarabaeoides*, AKPR375*- C. cajan.*

The IR regions are resistant to recombinational loss and therefore help in the stabilization of the cp genome (Perry and Wolfe, 2002). Both *C. scarabaeoides* and *C. cajan* possesses the smallest IR among the legumes and includes 21 completely duplicated genes. At IR/LSC junction *rps19* gene is excluded from the IR, rather *rpl2* gene is included and hence the whole *rpl2* gene is duplicated and included in the IR. Subsequently the IR merges into *ycf1* gene at IR/SSC junction with 448 bp and 444 bp of *ycf1* gene included in the IR region of *C. scarabaeoides* and *C. cajan* respectively. On comparing the cp genomes of *C. scarabaeoides* and *C. cajan* with other legumes it was observed that *rps19* gene (68 bp) was included in the IR region of *G. max* and showed partial duplication while in *V. radiata* and *P. vulgaris* the complete *rps19* gene was included and hence duplicated in the IR region. This feature however varies between the legumes as *rps19* gene is absent from the IR of *Millettia* and *Lupinus* (Williams et al., 2015), which is similar to that observed in pigeonpea. On the other hand, at IR/SSC junction, the *ycf1* gene is included in the IR in all the legumes but to different extents (Figure 5). Absence of *rps19* gene from the IR of pigeonpea plastid genome makes it smallest among all legumes leading to a bigger SSC region. This phenomenon of IR expansion and contraction could have resulted into the size variation among the legume cp genomes.

### RNA editing sites in transcripts from *C. scarabaeoides* and *C. cajan*

Editing sites in the cp DNA of pigeonpea genotypes were identified by PREP-cp program. It predicted 63 editing sites in 23 genes in *C. scarabaeoides* and 62 editing sites in 22 genes in *C. cajan*. Validation of the editing sites was done by mapping the transcriptome reads onto the DNA sequences from chloroplast and the sites having minimum 5X coverage were considered. Confirmation of editing at 37 sites in *C. scarabaeoides* and *C. cajan* was observed. In addition, 8 editing sites in *C. scarabaeoides* and *C. cajan* were identified, which were not predicted by PREPcp. Among all the genes analyzed, *ndh* gene displays the maximum number of editing sites (Supplementary Tables S6, S7), *ndh* genes have been reported to contain maximum number of editing sites (Corneille et al., 2000; Huang et al., 2013), since they are considered to be dispensable (Burrows et al., 1998; Shikanai et al., 1998), therefore accumulation of editing sites may have been permitted in *ndh* transcript due to dearth of stringent requirement of *ndh* function.

The editing type observed was 100% C–U, out of which 13.5% were silent, and 86.4% non-silent in both *C. scarabaeoides* and *C. cajan*. Silent editing occurs due to change in the third codon position which therefore does not lead to any amino acid change (Maier et al., 1996). Though silent RNA editing is frequent in mitochondrial genome which could account for 30% but it was reported for the first time in tobacco chloroplast genome at only one site in *atpA* gene (Hirose et al., 1996).

The editing event was most frequent at 2nd codon position in pigeonpea cp genome with 78.3% of editing occurring at 2nd position in both *C. scarabaeoides* and *C. cajan*. Among the amino acid changes 23 were converted from hydrophilic to hydrophobic and 1 amino acid from hydrophobic to hydrophilic in *C. scarabaeoides*. Similarly, 23 amino acids were converted from hydrophilic to hydrophobic and 1 amino acid from hydrophobic to hydrophilic in *C. cajan*. In both the genomes maximum conversion was observed for serine to leucine (45.9%). As evident from the results editing changes lead to increased number of hydrophobic amino acids as compared to hydrophilic amino acids in both the genotypes. These results are consistent with findings in other cp genomes also (Lee et al., 2014; Raman and Park, 2015). This bias might reflect the codon usage of plant plastome or may be the result of constraints due to the editing mechanism. For example, amino acid leucine may be preferred as it is a hydrophobic amino acid therefore prefers to be buried in the protein hydrophobic cores and hence involved in binding/recognition of hydrophobic ligands such as lipids.

Generally, the editing occurs in protein coding regions of chloroplast to restore the evolutionary conserved amino acids sequence (Maier et al., 1996). Like in pigeonpea cp genome, the frequency of editing sites is similar to that observed in other legumes like Pea (Inada et al., 2004) and *V. radiata* (Lin et al., 2015). Generally the editing sites vary between 20 and 37 in angiosperms (Hirose et al., 1999; Corneille et al., 2000; Lutz and Maliga, 2001). On the basis of comparison of editing frequencies and patterns it has been predicted that RNA editing is specific to a particular species. Although, editing has been found in all major lineages of land plants but its pattern does not correspond to the position of a particular species in the phylogenetic tree (Freyer et al., 1997).

### Microsatellite mining

**Figure 6:**
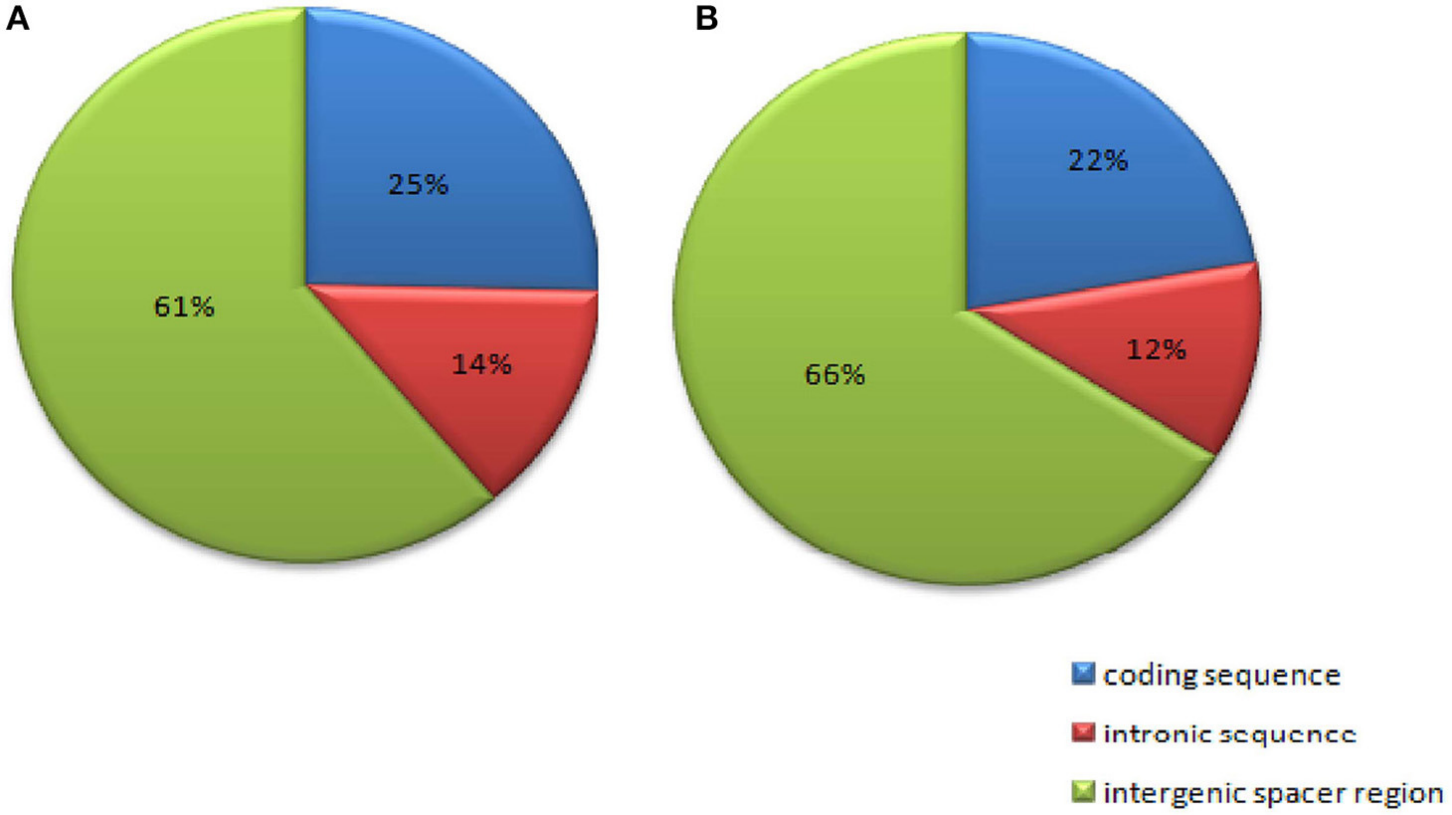
**Repeat distribution among three different regions: coding sequences, intronic sequences, and intergenic spacer regions (A)** AKPA1 (*C. scarabaeoides*); **(B)** AKPR375 (*C. cajan)*.

Chloroplast microsatellites (cpSSRs) are highly polymorphic due to the conserved gene order, non-recombinant and uniparentally inherited nature of the chloroplast genome (cpDNA) making them useful tools for studying phylogenetic relationships in plants (Olmstead and Palmer, 1994). We analyzed chloroplast SSRs (cpSSRs) with the MISA perl script and a total of 280 and 292 cpSSRs were identified in *C. scarabaeoides* and *C. cajan* respectively. The number was higher than that of cpSSRs identified in *V. radiata, Sesamum indicum* and *Camellia* species (Yi and Kim, 2012; Huang et al., 2014; Lin et al., 2015). Of the 280 repeats identified in *C. scarabaeoides*: 71.07% (199 SSRs) were located in the LSC region, 17.85% (50 SSRs) in the SSC region and 31% (11.07 SSRs) in the IR regions. In contrast, out of the 292 repeat motifs identified in *C. cajan*, 72.26% (211 SSRs) were present in LSC region, 17.46% (51 SSRs) in the SSC region and the remaining 10.27% (30 SSRs) were located in the IR regions, as reported in other plants like olives and artichoke (Mariotti et al., 2010; Curci et al., 2015). Furthermore, the SSR repeats were distributed among three different regions: coding sequence, intronic sequence, and intergenic spacer regions (Figure 6). 171 (61%) and 193 (66%) SSRs were located in the intergenic spacer regions of *C. scarabaeoides* and *C. cajan* respectively. Followed by 71 (25%) and 65 (22%) SSRs in the coding sequence and the remaining 38 (14%) and 34 (12%) repeats were present in the intronic regions. These results were in accordance with those reported in *G. max* (Ozyigit et al., 2015) indicating high degree of homology and conserved nature of genomes.

Of the SSRs identified, 49.28% (138 SSRs) and 45.89% (134 SSRs) were perfect repeats in *C. scarabaeoides* and *C. cajan* respectively. While 5.7%, 0.35% and 14.6% SSRs constituted imperfect, compound and compound imperfect repeats in *C. scarabaeoides* and 6.16%, 0.3%, 15.41% SSRs in *C. cajan* respectively.

**Figure 7:**
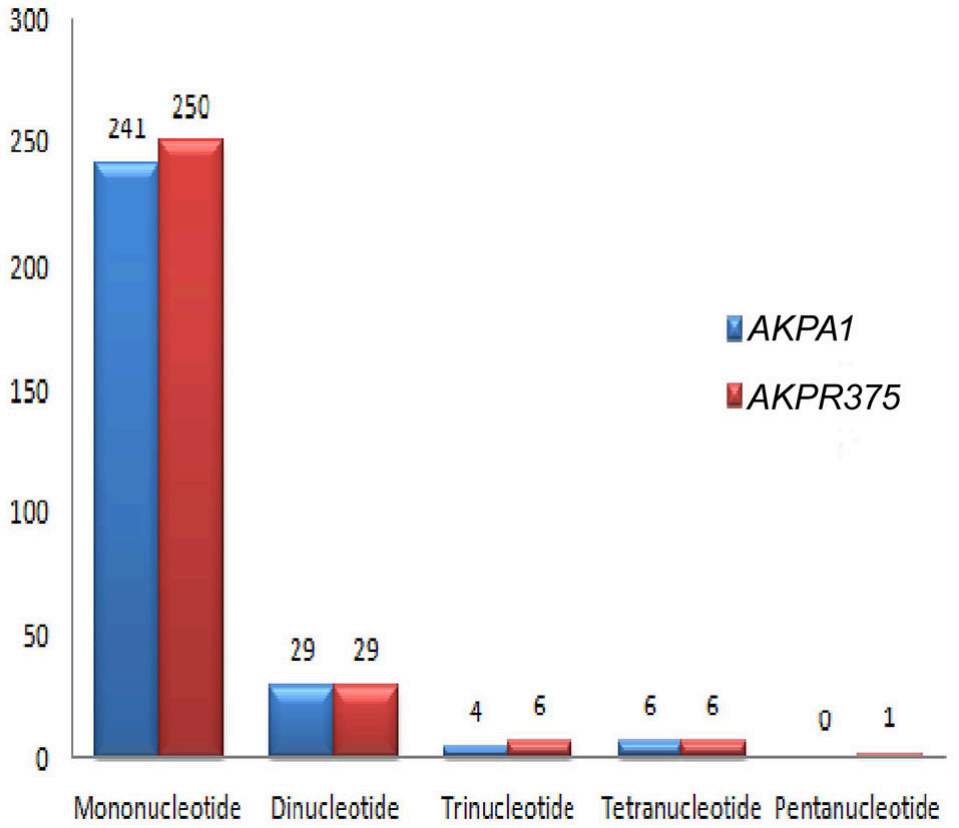
**SSR distribution on the basis of repeat type**.

**Figure 8:**
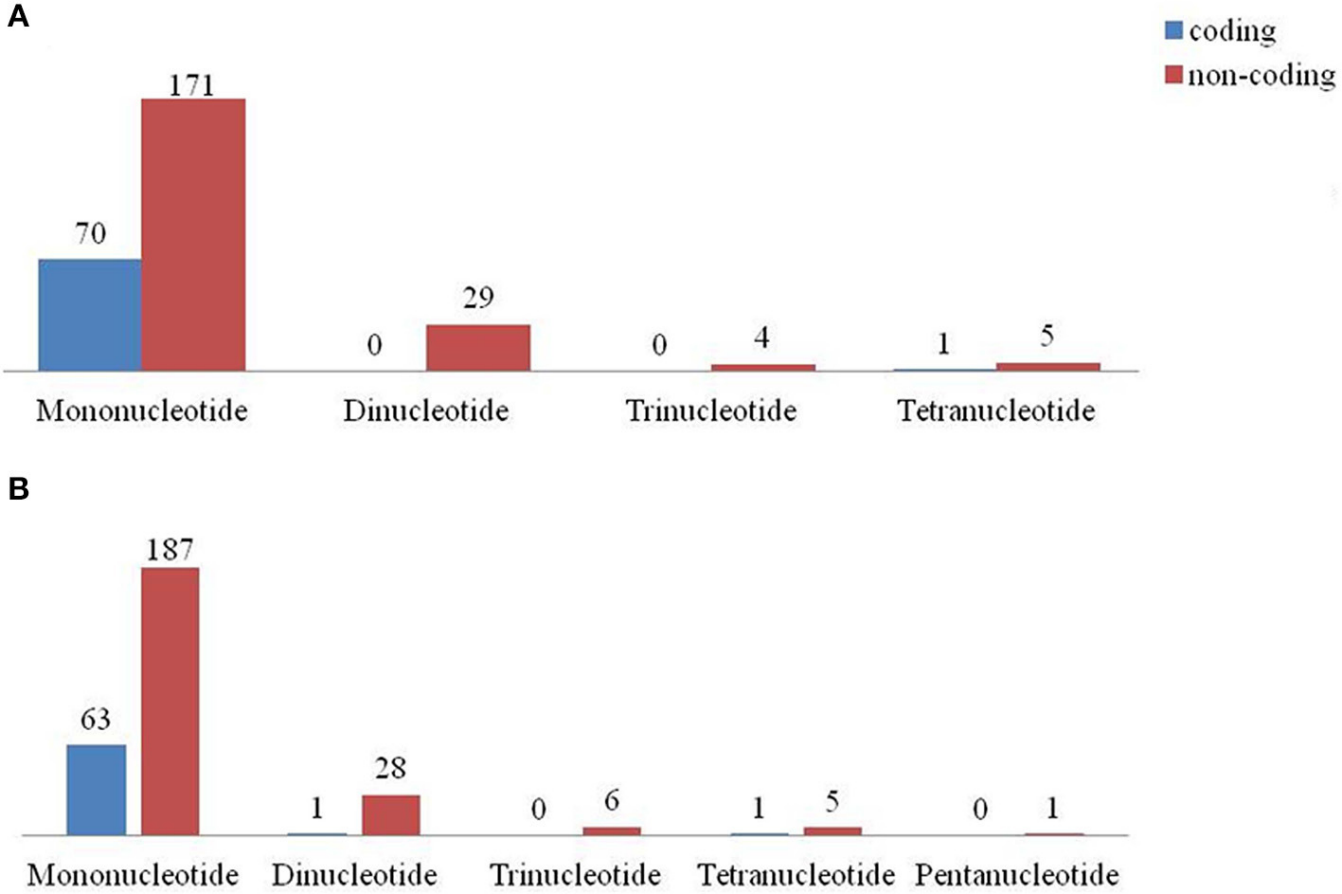
**SSR type distribution between coding and non-coding regions (A)** AKPA1 (*C. scarabaeoides*); **(B)** AKPR375 (*C. cajan)*.

Among the repeat types, the most abundant repeat was found to be mononucleotides in both *C. scarabaeoides* and *C. cajan* (Figure 7), with no hexa- repeats identified in both the genotypes and were distributed among the coding and non-coding regions (Figures 8A,B). The findings were in agreement with those in *Sesame* (Yi and Kim, 2012) and olive species (Mariotti et al., 2010). Majority of the microsatellites in the chloroplast genome are mononucleotide A/T repeats (Wheeler et al., 2014). Likewise, mononucleotides A/T were predominant in both pigeonpea genotypes which is in agreement with results from previous studies in *Oryza sativa, V. radiata, Camellia* species and *Sesame indicum* (Rajendrakumar et al., 2007; Tangphatsornruang et al., 2010; Yi and Kim, 2012; Huang et al., 2014). AT/TA (93.10%) was most frequent dinucleotide motifs followed by AG/TC in both *C. scarabaeoides* and *C. cajan* respectively. Higher frequency of AT/TA motifs was also reported in *Glycine* species, olive species and *Sesamum indicum* (Mariotti et al., 2010; Yi and Kim, 2012; Ozyigit et al., 2015). AAT/TTA and AAAT/TTTA were the most frequent trinucleotide and tetranucleotide motifs followed by ATT/TAA and AATA/ TTAT in both *C. scarabaeoides* and *C. cajan*. Only one pentanucleotide motif TATTA/ATAAT was identified in the *C. cajan*, while no hexameric repeats were observed. This is evident from the AT bias the plastid genomes seems to possess.

The chloroplast gene possessing the highest number of repeats was *ycf1* in both the genotypes (Supplementary Tables S8, S9). Our findings are in agreement with those from *Glycine* species, *V. radiata, Camellia* species, *Cynara cardunculus* (Tangphatsornruang et al., 2010; Huang et al., 2014; Curci et al., 2015; Ozyigit et al., 2015). Dong et al. (2012) expressed *ycf1* gene as the most variable locus accordingly highly variable SSRs can be located in the *ycf1* coding region of the pigeonpea cp genome.

## Conclusion

The draft cp genome of *C. scarabaeoides* and *C. cajan* were sequenced by Roche-454 technology. This is the first study reporting the sequence of pigeonpea cp genome. The pigeonpea cp genome is similar to other legume cp genomes, in terms of cp genome size and number of unique genes. The organization of pigeonpea cp genome shows similarity to other legume cp genomes except for IR contraction and hence exclusion of *rps19* gene from the IR. The genes *rps16, rpl33, ycf15, ycf68*, and *ycf1* were observed as pseudogenes and *rpl22* and *infA* are absent from the pigeonpea cp genome. RNA editing was also observed at 37 sites in both plastids, particularly in *ndh* gene region. Chloroplast SSRs were also mined, with 280 and 292 cpSSRs being identified in *C. scarabaeoides* and *C. cajan* respectively. This study would be helpful in phylogenetic and evolutionary studies of pigeonpea with other legumes.

## Author contributions

TK carried out the experiments, prepared the genomic library for Roche sequencing and sequencing run and wrote the manuscript. PC performed chloroplast genome assembly and bioinformatics analysis. SS carried out the SSR markers discovery and validation. TK, PC, SS, and KB were involved in the result interpretation, analysis, and finalization of the manuscript. NS, TS, and AC contributed in data analysis, genome annotation, and manuscript finalization. SG provided the germplasm and assisted in data analysis. KG conceived the study, designed the experiments, and coordinated the work. All the authors have read and approved the final manuscript.

### Conflict of interest statement

The authors declare that the research was conducted in the absence of any commercial or financial relationships that could be construed as a potential conflict of interest.

## Footnotes

^1^https://www.ncbi.nlm.nih.gov/genome/browse/

^2^http://www.bioinformatics.babraham.ac.uk/projects/fastqc

^3^http://www.currentprotocols.com/WileyCDA/CurPro3Tool/toolId-7.html

^4^http://prep.unl.edu/

^5^http://pgrc.ipk-gatersleben.de/misa/
