# Supplementary material for: Chloroplast Genome Sequence of Pigeonpea (Cajanus cajan (L.) Millspaugh) and Cajanus scarabaeoides (L.) Thouars: Genome Organization and Comparison with Other Legumes
Source: Front Plant Sci. 2016 Dec 9;7:1847. doi: 10.3389/fpls.2016.01847 (PMC5145887; doi:10.3389/fpls.2016.01847)
Supplement: Supplementary file 1 [file Table1.DOCX]

**Supplementary Table S1- Size comparison among six cp genomes completely sequenced in legumes**

| **Species** | **Accession Number** | **Genome size (bp)** | **LSC (bp)** | **SSC (bp)** | **IR (bp)** | **No. of unique genes** | **No. of inverted repeats** |
| --- | --- | --- | --- | --- | --- | --- | --- |
| *Glycine max* | NC_007942 | 152,218 | 83,175 | 17,895 | 25,574 | 111 | 2 |
| *Phaseolus vulgaris* | [NC_009259](http://www.ncbi.nlm.nih.gov/nuccore/NC_009259) | 150,285 | 79,824 | 17,610 | 26,426 | 108 | 2 |
| *Vigna radiata* | [NC_013843](http://www.ncbi.nlm.nih.gov/nuccore/NC_013843) | 151, 271 | 80,896 | 17,427 | 26,474 | 108 | 2 |
| *Cicer arietinum* | NC_011163 | 125,319 | - | - | 50,000 | 108 | 1 |
| *Cajanus scarabaeoides* | KU729878 | 152,201 | 83,423 | 17,854 | 25,402 | 116 | 2 |
| *Cajanus cajan* | KU729879 | 152,242 | 83,455 | 17,871 | 25,398 | 116 | 2 |
