# Supplementary material for: Chloroplast Genome Sequence of Pigeonpea (Cajanus cajan (L.) Millspaugh) and Cajanus scarabaeoides (L.) Thouars: Genome Organization and Comparison with Other Legumes
Source: Front Plant Sci. 2016 Dec 9;7:1847. doi: 10.3389/fpls.2016.01847 (PMC5145887; doi:10.3389/fpls.2016.01847)
Supplement: Supplementary file 2 [file Table2.DOCX]

**Supplementary Table S2- Codon Usage for *Cajanus scarabaeoides***

| **Amino acid** | **Codon** | **Count** | **RSCU** | **tRNA** |
| --- | --- | --- | --- | --- |
| Phe | UUU | 1100 | 1.30 |  |
| Phe | UUC | 598 | 0.70 | trnF-GAA |
| Leu | UUA | 881 | 1.82 | trnL-UAA |
| Leu | UUG | 600 | 1.24 | trnL-CAA |
| Leu | CUU | 573 | 1.19 |  |
| Leu | CUC | 213 | 0.44 |  |
| Leu | CUA | 428 | 0.89 | trnL-UAG |
| Leu | CUG | 203 | 0.42 |  |
| Ile | AUU | 1140 | 1.40 |  |
| Ile | AUC | 486 | 0.60 | trnI-GAU |
| Ile | AUA | 812 | 1.00 |  |
| Met | AUG | 600 | 1.00 | trnM-CAU |
| Val | GUU | 522 | 1.46 |  |
| Val | GUC | 176 | 0.49 | trnV-GAC |
| Val | GUA | 524 | 1.46 | trnV-UAC |
| Val | GUG | 210 | 0.59 |  |
| Ser | UCU | 524 | 1.69 |  |
| Ser | UCC | 281 | 0.91 | trnS-GGA |
| Ser | UCA | 407 | 1.31 | trnS-UGA |
| Ser | UCG | 187 | 0.60 |  |
| Pro | CCU | 360 | 1.42 |  |
| Pro | CCC | 198 | 0.78 | trnP-GGG |
| Pro | CCA | 317 | 1.25 | trnP-UGG |
| Pro | CCG | 141 | 0.56 |  |
| Thr | ACU | 473 | 1.57 |  |
| Thr | ACC | 205 | 0.68 | trnT-GGU |
| Thr | ACA | 395 | 1.31 | trnT-UGU |
| Thr | ACG | 130 | 0.43 |  |
| Ala | GCU | 513 | 1.79 |  |
| Ala | GCC | 172 | 0.60 |  |
| Ala | GCA | 347 | 1.21 | trnA-UGC |
| Ala | GCG | 113 | 0.39 |  |
| Tyr | UAU | 840 | 1.55 |  |
| Tyr | UAC | 246 | 0.45 | trnY-GUA |
| TER | UAA | 207 | 1.17 |  |
| TER | UAG | 172 | 0.97 |  |
| His | CAU | 477 | 1.51 |  |
| His | CAC | 156 | 0.49 | trnH-GUG |
| Gln | CAA | 737 | 1.49 | trnQ-UUG |
| Gln | CAG | 252 | 0.51 |  |
| Asn | AAU | 955 | 1.53 |  |
| Asn | AAC | 297 | 0.47 | trnN-GUU |
| Lys | AAA | 1167 | 1.52 | trnK-UUU |
| Lys | AAG | 370 | 0.48 |  |
| Asp | GAU | 717 | 1.58 |  |
| Asp | GAC | 191 | 0.42 | trnD-GUC |
| Glu | GAA | 938 | 1.51 | trnE-UUC |
| Glu | GAG | 303 | 0.49 |  |
| Cys | UGU | 248 | 1.40 |  |
| Cys | UGC | 106 | 0.60 | trnC-GCA |
| TER | UGA | 154 | 0.87 |  |
| Trp | UGG | 461 | 1.00 | trnW-CCA |
| Arg | CGU | 280 | 1.13 | trnR-ACG |
| Arg | CGC | 88 | 0.35 |  |
| Arg | CGA | 338 | 1.36 |  |
| Arg | CGG | 135 | 0.54 |  |
| Ser | AGU | 344 | 1.11 |  |
| Ser | AGC | 115 | 0.37 | trnS-GCU |
| Arg | AGA | 484 | 1.95 | trnR-UCU |
| Arg | AGG | 168 | 0.68 |  |
| Gly | GGU | 523 | 1.33 | trnG-UCC |
| Gly | GGC | 156 | 0.40 |  |
| Gly | GGA | 613 | 1.56 |  |
| Gly | GGG | 283 | 0.72 |  |

RSCU- relative synonymous codon usage
