# Supplementary material for: Chloroplast Genome Sequence of Pigeonpea (Cajanus cajan (L.) Millspaugh) and Cajanus scarabaeoides (L.) Thouars: Genome Organization and Comparison with Other Legumes
Source: Front Plant Sci. 2016 Dec 9;7:1847. doi: 10.3389/fpls.2016.01847 (PMC5145887; doi:10.3389/fpls.2016.01847)
Supplement: Supplementary file 3 [file Table3.DOCX]

**Supplementary Table S3- Codon Usage for *Cajanus cajan***

| **Amino acid** | **Codon** | **Count** | **RSCU** | **tRNA** |
| --- | --- | --- | --- | --- |
| Phe | UUU | 1123 | 1.35 |  |
| Phe | UUC | 541 | 0.65 | trnF-GAA |
| Leu | UUA | 751 | 1.99 | trnL-UAA |
| Leu | UUG | 472 | 1.25 | trnL-CAA |
| Leu | CUU | 475 | 1.26 |  |
| Leu | CUC | 145 | 0.38 |  |
| Leu | CUA | 283 | 0.75 | trnL-UAG |
| Leu | CUG | 138 | 0.37 |  |
| Ile | AUU | 1080 | 1.53 |  |
| Ile | AUC | 414 | 0.59 | trnI-GAU |
| Ile | AUA | 627 | 0.89 |  |
| Met | AUG | 463 | 1.00 | trnM-CAU |
| Val | GUU | 490 | 1.57 |  |
| Val | GUC | 163 | 0.52 | trnV-GAC |
| Val | GUA | 441 | 1.41 | trnV-UAC |
| Val | GUG | 154 | 0.49 |  |
| Ser | UCU | 586 | 1.67 |  |
| Ser | UCC | 330 | 0.94 | trnS-GGA |
| Ser | UCA | 427 | 1.22 | trnS-UGA |
| Ser | UCG | 194 | 0.55 |  |
| Pro | CCU | 347 | 1.48 |  |
| Pro | CCC | 182 | 0.77 | trnP-GGG |
| Pro | CCA | 292 | 1.24 | trnP-UGG |
| Pro | CCG | 119 | 0.51 |  |
| Thr | ACU | 472 | 1.52 |  |
| Thr | ACC | 252 | 0.81 | trnT-GGU |
| Thr | ACA | 385 | 1.24 | trnT-UGU |
| Thr | ACG | 137 | 0.44 |  |
| Ala | GCU | 515 | 1.81 |  |
| Ala | GCC | 163 | 0.57 |  |
| Ala | GCA | 345 | 1.21 | trnA-UGC |
| Ala | GCG | 116 | 0.41 |  |
| Tyr | UAU | 863 | 1.55 |  |
| Tyr | UAC | 248 | 0.45 | trnY-GUA |
| TER | UAA | 154 | 1.00 |  |
| TER | UAG | 100 | 0.65 |  |
| His | CAU | 482 | 1.56 |  |
| His | CAC | 134 | 0.44 | trnH-GUG |
| Gln | CAA | 647 | 1.56 | trnQ-UUG |
| Gln | CAG | 180 | 0.44 |  |
| Asn | AAU | 1018 | 1.55 |  |
| Asn | AAC | 293 | 0.45 | trnN-GUU |
| Lys | AAA | 1068 | 1.54 | trnK-UUU |
| Lys | AAG | 317 | 0.46 |  |
| Asp | GAU | 742 | 1.59 |  |
| Asp | GAC | 190 | 0.41 | trnD-GUC |
| Glu | GAA | 903 | 1.52 | trnE-UUC |
| Glu | GAG | 286 | 0.48 |  |
| Cys | UGU | 268 | 1.29 |  |
| Cys | UGC | 148 | 0.71 | trnC-GCA |
| TER | UGA | 207 | 1.35 |  |
| Trp | UGG | 430 | 1.00 | trnW-CCA |
| Arg | CGU | 297 | 1.14 | trnR-ACG |
| Arg | CGC | 87 | 0.33 |  |
| Arg | CGA | 327 | 1.25 |  |
| Arg | CGG | 116 | 0.44 |  |
| Ser | AGU | 391 | 1.12 |  |
| Ser | AGC | 172 | 0.49 | trnS-GCU |
| Arg | AGA | 527 | 2.02 | trnR-UCU |
| Arg | AGG | 213 | 0.82 |  |
| Gly | GGU | 504 | 1.28 | trnG-UCC |
| Gly | GGC | 164 | 0.42 |  |
| Gly | GGA | 619 | 1.57 |  |
| Gly | GGG | 293 | 0.74 |  |

RSCU- relative synonymous codon usage
