# Supplementary material for: Chloroplast Genome Sequence of Pigeonpea (Cajanus cajan (L.) Millspaugh) and Cajanus scarabaeoides (L.) Thouars: Genome Organization and Comparison with Other Legumes
Source: Front Plant Sci. 2016 Dec 9;7:1847. doi: 10.3389/fpls.2016.01847 (PMC5145887; doi:10.3389/fpls.2016.01847)
Supplement: Supplementary file 4 [file Table4.docx]

**Supplementary Table S4- List of genes with introns in cp genome of *Cajanus scarabaeoides***

| S. No | Gene | Strand | Region | Start | End | Exon I (bp) | Intron I (bp) | Exon II (bp) | Intron II (bp) | Exon III (bp) |
| --- | --- | --- | --- | --- | --- | --- | --- | --- | --- | --- |
| 1 | *trnK-UUU* | - | LSC | 1656 | 4313 | 28 | 2593 | 36 | - | - |
| 2 | *trnV-UAC* | + | LSC | 9965 | 10635 | 38 | 597 | 34 | - | - |
| 3 | *trnL-UAA* | - | LSC | 13687 | 14290 | 49 | 518 | 36 | - | - |
| 4 | *ycf3* | + | LSC | 16626 | 18613 | 125 | 694 | 227 | 789 | 152 |
| 5 | *rpoC1* | + | LSC | 37800 | 40661 | 440 | 790 | 1628 | - | - |
| 6 | *atpF* | + | LSC | 48754 | 50047 | 167 | 716 | 407 | - | - |
| 7 | *clpP* | - | LSC | 68744 | 70788 | 227 | 748 | 299 | 702 | 68 |
| 8 | *rpl2* | - | IR a | 83423 | 84932 | 434 | 680 | 395 | - | - |
| 9 | *ycf2* | + | IR a | 85339 | 92747 | 302 | 198 | 6908 | - | - |
| 10 | *ndhB* | - | IR a | 93817 | 96012 | 755 | 664 | 776 | - | - |
| 11 | *trnI-GAU* | + | IR a | 101499 | 102525 | 41 | 951 | 34 | - | - |
| 12 | *trnA-UGC* | + | IR a | 102590 | 103473 | 37 | 812 | 34 | - | - |
| 13 | *trnA-UGC* | - | IR b | 132036 | 132919 | 37 | 812 | 34 | - | - |
| 14 | *trnI-GAU* | - | IR b | 132984 | 134010 | 41 | 951 | 34 | - | - |
| 15 | *ndhB* | + | IR b | 139497 | 141692 | 755 | 664 | 776 | - | - |
| 16 | *ycf2* | - | IR b | 142762 | 150170 | 302 | 198 | 6908 | - | - |
| 17 | *rpl2* | + | IR b | 150577 | 152086 | 434 | 680 | 395 | - | - |
