# Supplementary material for: Chloroplast Genome Sequence of Pigeonpea (Cajanus cajan (L.) Millspaugh) and Cajanus scarabaeoides (L.) Thouars: Genome Organization and Comparison with Other Legumes
Source: Front Plant Sci. 2016 Dec 9;7:1847. doi: 10.3389/fpls.2016.01847 (PMC5145887; doi:10.3389/fpls.2016.01847)
Supplement: Supplementary file 5 [file Table5.DOCX]

**Supplementary Table S5- List of genes with introns in cp genome of *Cajanus cajan***

| S. No | Gene | Strand | Region | Start | End | Exon I (bp) | Intron I (bp) | Exon II (bp) | Intron II (bp) | Exon III (bp) |
| --- | --- | --- | --- | --- | --- | --- | --- | --- | --- | --- |
| 1 | *trnK-UUU* | - | LSC | 1649 | 4307 | 28 | 2594 | 36 | - | - |
| 2 | *trnV-UAC* | + | LSC | 9958 | 10628 | 38 | 598 | 34 | - | - |
| 3 | *trnL-UAA* | - | LSC | 13681 | 14287 | 49 | 521 | 36 | - | - |
| 4 | *ycf3* | + | LSC | 16621 | 18607 | 125 | 694 | 227 | 777 | 149 |
| 5 | *rpoC1* | + | LSC | 37808 | 40669 | 440 | 790 | 1628 | - | - |
| 6 | *atpF* | + | LSC | 48770 | 50063 | 167 | 716 | 407 | - | - |
| 7 | *clpP* | - | LSC | 68766 | 70810 | 227 | 748 | 299 | 702 | 68 |
| 8 | *rpl2* | - | IR a | 83455 | 84964 | 434 | 680 | 395 | - | - |
| 9 | *ycf2* | + | IR a | 85371 | 92779 | 302 | 198 | 6908 | - | - |
| 10 | *ndhB* | - | IR a | 93849 | 96042 | 755 | 662 | 776 | - | - |
| 11 | *trnI-GAU* | + | IR a | 101531 | 102557 | 41 | 951 | 34 | - | - |
| 12 | *trnA-UGC* | + | IR a | 102622 | 103504 | 37 | 811 | 34 | - | - |
| 13 | *trnA-UGC* | - | IR b | 132078 | 132960 | 37 | 811 | 34 | - | - |
| 14 | *trnI-GAU* | - | IR b | 133025 | 134051 | 41 | 951 | 34 | - | - |
| 15 | *ndhB* | + | IR b | 139540 | 141733 | 755 | 662 | 776 | - | - |
| 16 | *ycf2* | - | IR b | 142803 | 150211 | 302 | 198 | 6908 | - | - |
| 17 | *rpl2* | + | IR b | 150618 | 152127 | 434 | 680 | 395 | - | - |
