# Supplementary material for: Chloroplast Genome Sequence of Pigeonpea (Cajanus cajan (L.) Millspaugh) and Cajanus scarabaeoides (L.) Thouars: Genome Organization and Comparison with Other Legumes
Source: Front Plant Sci. 2016 Dec 9;7:1847. doi: 10.3389/fpls.2016.01847 (PMC5145887; doi:10.3389/fpls.2016.01847)
Supplement: Supplementary file 6 [file Table6.DOCX]

**Supplementary Table S6- RNA Editing in *Cajanus scarabaeoides***

| S.No. | Gene Name | Editing Change | Amino acid Change | Nucleotide Position | Codon Position | Codon Change |
| --- | --- | --- | --- | --- | --- | --- |
| 1 | *accD* | C-T | S-L | 584 | 2 | TCG-TTG |
| 2 | *atpA* | C-T | P-L | 791 | 2 | CCC-CTG |
| 3 | *atpF* | C-T | P-L | 92 | 2 | CCA-CTA |
| 4 | *ndhB* | C-T | S-L | 149 | 2 | TCA-TTA |
|  |  | C-T | F-F | 273 | 3 | TTC-TTT |
|  |  | C-T | T-M | 542 | 2 | ACG-ATG |
|  |  | C-T | H-Y | 586 | 1 | CAT-TAT |
|  |  | C-T | P-L | 737 | 2 | CCA-CTA |
|  |  | C-T | S-F | 746 | 2 | TCT-TTT |
|  |  | C-T | S-L | 1493 | 2 | TCA-TTA |
|  |  | C-T | S-L | 1499 | 2 | TCA-TTA |
|  |  | C-T | S-L | 1775 | 2 | TCA-TTA |
|  |  | C-T | H-Y | 1918 | 1 | CAT-TAT |
| 5 | *ndhD* | C-T | T-I | 380 | 2 | ACA-ATA |
|  |  | C-T | S-L | 875 | 2 | TCA-TTA |
|  |  | C-T | S-L | 1295 | 2 | TCA-TTA |
| 6 | *petB* | C-T | V-V | 12 | 3 | GTC-GTT |
|  |  | C-T | S-L | 611 | 2 | TCA-TTA |
| 7 | *psbE* | C-T | P-S | 214 | 1 | CCT-TCT |
| 8 | *psbF* | C-T | S-F | 77 | 2 | TCT-TTT |
| 9 | *rpoB* | C-T | F-F | 338 | 3 | TTC-TTT |
|  |  | C-T | S-L | 5511 | 2 | TCA-TTA |
|  |  | C-T | F-F | 5661 | 3 | TTC-TTT |
| 10 | *rpoC1* | C-T | S-L | 41 | 2 | TCA-TTA |
| 11 | *rpoC2* | C-T | S-L | 3740 | 2 | TCG-TTG |
| 12 | *rps2* | C-T | S-L | 248 | 2 | TCA-TTA |
| 13 | *rps14* | C-T | S-L | 80 | 2 | TCA-TTA |
| 14 | *rps16* | C-T | S-L | 176 | 2 | TCA-TTA |
| 15 | *ndhA* | C-T | S-F | 524 | 2 | TCT-TTT |
| 16 | *rpl23* | C-T | S-L | 89 | 2 | TCA-TTA |
| 17 | *ndhE* | C-T | P-L | 233 | 2 | CCG-CTG |
| 18 | *ndhC* | C-T | S-L | 323 | 2 | TCA-TTA |
| 19 | *ndhK* | C-T | F-F | 81 | 3 | TTC-TTT |
| 20 | *petL* | C-T | S-F | 5 | 2 | TCC-TTC |
| 21 | *psbN* | C-T | S-F | 29 | 2 | TCT-TTT |
| 22 | *rps18* | C-T | S-L | 221 | 2 | TCG-TTG |
| 23 | *psbJ* | C-T | P-L | 59 | 2 | CCT-CTT |
