# Supplementary material for: Chloroplast Genome Sequence of Pigeonpea (Cajanus cajan (L.) Millspaugh) and Cajanus scarabaeoides (L.) Thouars: Genome Organization and Comparison with Other Legumes
Source: Front Plant Sci. 2016 Dec 9;7:1847. doi: 10.3389/fpls.2016.01847 (PMC5145887; doi:10.3389/fpls.2016.01847)
Supplement: Supplementary file 7 [file Table7.DOCX]

**Supplementary Table S7- RNA Editing in *Cajanus cajan***

| S.No. | Gene Name | Editing Change | Amino acid Change | Nucleotide Position | Codon Position | Codon change |
| --- | --- | --- | --- | --- | --- | --- |
| 1 | *accD* | C-T | S-L | 584 | 2 | TCG-TTG |
| 2 | *atpA* | C-T | P-L | 791 | 2 | CCC-CTC |
| 3 | *clpP* | C-T | S-L | 1766 | 2 | TCA-TTA |
|  |  | C-T | H-Y | 2014 | 1 | CAC-TAC |
| 4 | *ndhA* | C-T | S-F | 525 | 2 | TCT-TTT |
| 5 | *ndhB* | C-T | S-L | 150 | 2 | TCA-TTA |
|  |  | C-T | F-F | 274 | 3 | TTC-TTT |
|  |  | C-T | T-M | 543 | 2 | ACG-ATG |
|  |  | C-T | H-Y | 587 | 1 | CAT-TAT |
|  |  | C-T | P-L | 738 | 2 | CCA-CTA |
|  |  | C-T | S-F | 747 | 2 | TCT-TTT |
|  |  | C-T | S-L | 1492 | 2 | TCA-TTA |
|  |  | C-T | S-L | 1498 | 2 | TCA-TTA |
|  |  | C-T | I-I | 1774 | 3 | ATC-ATT |
|  |  | C-T | P-L | 1917 | 2 | TCA-TTA |
| 6 | *ndhD* | C-T | T-I | 381 | 2 | ACA-ATA |
|  |  | C-T | S-L | 875 | 2 | TCA-TTA |
|  |  | C-T | S-L | 1295 | 2 | TCA-TTA |
| 7 | *petB* | C-T | S-F | 13 | 2 | TCT-TTT |
|  |  | C-T | S-L | 612 | 2 | TCA-TTA |
| 8 | *petL* | C-T | S-F | 6 | 2 | TCC-TTC |
| 9 | *psbE* | C-T | P-S | 215 | 1 | CCT-TCT |
| 10 | *psbF* | C-T | S-F | 78 | 2 | TCT-TTT |
| 11 | *rpl23* | C-T | S-L | 90 | 2 | TCA-TTA |
| 12 | *rpoB* | C-T | S-L | 566 | 2 | TCA-TTA |
|  |  | C-T | S-L | 2735 | 2 | TCG-TTG |
|  |  | C-T | P-P | 2757 | 3 | CCC-CCT |
| 13 | *rpoC2* | C-T | S-S | 2634 | 3 | TCC-TCT |
|  |  | C-T | S-L | 3740 | 2 | TCG-TTG |
| 14 | *rps2* | C-T | S-L | 248 | 2 | TCA-TTA |
| 15 | *rsp14* | C-T | S-L | 80 | 2 | TCA-TTA |
| 16 | *rps16* | C-T | S-L | 176 | 2 | TCA-TTA |
| 17 | *ndhC* | C-T | S-L | 323 | 2 | TCA-TTA |
| 18 | *ndhE* | C-T | P-L | 234 | 2 | CCG-CTG |
| 19 | *ndhK* | C-T | F-F | 81 | 3 | TTC-TTT |
| 20 | *psbJ* | C-T | P-L | 60 | 2 | CCT-CTT |
| 21 | *rps18* | C-T | S-L | 222 | 2 | TCG-TTG |
