# Supplementary material for: Chloroplast Genome Sequence of Pigeonpea (Cajanus cajan (L.) Millspaugh) and Cajanus scarabaeoides (L.) Thouars: Genome Organization and Comparison with Other Legumes
Source: Front Plant Sci. 2016 Dec 9;7:1847. doi: 10.3389/fpls.2016.01847 (PMC5145887; doi:10.3389/fpls.2016.01847)
Supplement: Supplementary file 8 [file Table8.DOCX]

**Supplementary Table S8- SSRs found in *Cajanus scarabaeoides***

| **SSR No.** | **SSR motif** | **Size** | **SSR type** | **Start** | **End** | ***Location*** | **Genomic region** |
| --- | --- | --- | --- | --- | --- | --- | --- |
| 1 | (A)8 | 8 | p | 1940 | 1947 | *trnK(UUU) intron* | intron |
| 2 | (T)9 | 9 | p | 2291 | 2299 | *trnK(UUU) intron* | intron |
| 3 | (A)8 | 8 | p | 2833 | 2840 | *trnK(UUU) intron* | intron |
| 4 | (A)8 | 8 | p | 2958 | 2965 | *trnK(UUU) intron* | intron |
| 5 | (A)8 | 8 | p | 3484 | 3491 | *trnK(UUU) intron* | intron |
| 6 | (T)9N(A)9 | 44 | ci | 3758 | 3801 | *trnK(UUU) intron* | intron |
| 7 | (A)12 | 12 | p | 3980 | 3991 | *trnK(UUU) intron* | intron |
| 8 | (A)8 | 8 | p | 4358 | 4365 | *trnK(UUU)-rbcL* | spacer |
| 9 | (T)8 | 8 | p | 4876 | 4883 | *trnK(UUU)-rbcL* | spacer |
| 10 | (AAAT)3 | 12 | p | 5062 | 5073 | *trnK(UUU)-rbcL* | spacer |
| 11 | (AT)18 | 36 | p | 5241 | 5276 | *trnK(UUU)-rbcL* | spacer |
| 12 | (T)12N(A)9 | 84 | ci | 7091 | 7174 | *rbcL-atpB* | spacer |
| 13 | (A)10 | 10 | p | 7431 | 7440 | *rbcL-atpB* | spacer |
| 14 | (T)9N(T)9N(A)10 | 107 | ci | 7628 | 7734 | *atpB* | cds |
| 15 | (T)9 | 9 | p | 10015 | 10023 | *trnV(uAC) intron* | intron |
| 16 | (A)11 | 11 | p | 11250 | 11260 | *ndhK* | cds |
| 17 | (A)9 | 9 | p | 11993 | 12001 | *ndhK-ndhJ* | spacer |
| 18 | (TA)5N(A)8 | 32 | ci | 13074 | 13105 | *ndhJ-trnF(GAA)* | spacer |
| 19 | (T)8N(A)8 | 24 | ci | 13463 | 13486 | *trnF(GAA)-trnL(UAA)* | spacer |
| 20 | (T)11 | 11 | p | 14459 | 14469 | *trnL(UAA)-trnT(UGU)* | spacer |
| 21 | (A)9N(T)8 | 73 | ci | 15192 | 15264 | *trnT(UGU)-rps4* | spacer |
| 22 | (TA)6N(A)9N(A)9 | 81 | ci | 16760 | 16840 | *ycf3 intron 1* | intron |
| 23 | (AT)5N(A)11 | 52 | ci | 17734 | 17785 | *ycf3 intron 2* | intron |
| 24 | (AT)5 | 10 | p | 18168 | 18177 | *ycf3 intron 2* | intron |
| 25 | (T)8N(A)8N(A)9 | 42 | ci | 18340 | 18381 | *ycf3 intron 2* | intron |
| 26 | (TA)6 | 12 | p | 18677 | 18688 | *ycf3-psaA* | intron |
| 27 | (A)9 | 9 | p | 18839 | 18847 | *ycf3-psaA* | intron |
| 28 | (A)8 | 8 | p | 18961 | 18968 | *ycf3-psaA* | intron |
| 29 | (T)8 | 8 | p | 24225 | 24232 | *rps14-trnfM(CAU)* | spacer |
| 30 | (T)8 | 8 | p | 24495 | 24502 | *trnG(UCC)-IhbA* | spacer |
| 31 | (T)8N(TTA)4 | 53 | ci | 24608 | 24660 | *trnG(UCC)-IhbA* | spacer |
| 32 | (T)9N(TA)8 | 65 | ci | 24786 | 24850 | *trnG(UCC)-IhbA* | spacer |
| 33 | (T)8 | 8 | p | 25393 | 25400 | *IhbA-trnS(UGA)* | spacer |
| 34 | (A)8 | 8 | p | 25770 | 25777 | *trnS(UGA)-psbC* | spacer |
| 35 | (T)9N(T)10 | 21 | i | 28598 | 28618 | *psbD-trnT(GGU)* | spacer |
| 36 | (ATT)4N(A)8 | 36 | ci | 28830 | 28865 | *psbD-trnT(GGU)* | spacer |
| 37 | (T)9N(T)8N(A)8 | 163 | ci | 29386 | 29548 | *psbD-trnT(GGU)* | spacer |
| 38 | (T)14N(T)8 | 117 | i | 30195 | 30311 | *trnM(CAU)-trnE(UUC)* | spacer |
| 39 | (T)8 | 8 | p | 30725 | 30732 | *trnY(GUA)-trnD(GUC)* | spacer |
| 40 | (A)8N(A)8N(T)9 | 149 | ci | 31716 | 31864 | *psbM-petN* | spacer |
| 41 | (A)8 | 8 | p | 31982 | 31989 | *psbM-petN* | spacer |
| 42 | (T)9N(A)10 | 92 | ci | 32560 | 32651 | *petN-trnC(GCA)* | spacer |
| 43 | (T)8 | 8 | p | 32780 | 32787 | *petN-trnC(GCA)* | spacer |
| 44 | (T)8 | 8 | p | 32984 | 32991 | *petN-trnC(GCA)* | spacer |
| 45 | (T)9N(A)8 | 98 | ci | 33433 | 33530 | *petN-trnC(GCA)* | spacer |
| 46 | (T)8N(A)9 | 28 | ci | 33674 | 33701 | *trnC(GCA)-rpoB* | spacer |
| 47 | (A)9 | 9 | p | 33879 | 33887 | *trnC(GCA)-rpoB* | spacer |
| 48 | (AT)7N(T)10 | 62 | ci | 33989 | 34050 | *trnC(GCA)-rpoB* | spacer |
| 49 | (G)8 | 8 | p | 35021 | 35028 | *rpoB* | cds |
| 50 | (A)10N(A)10 | 114 | i | 35197 | 35310 | *rpoB* | cds |
| 51 | (A)8 | 8 | p | 37871 | 37878 | *rpoC1 exon1* | cds |
| 52 | (A)13 | 13 | p | 38460 | 38472 | *rpoC1 intron 1* | intron |
| 53 | (A)10 | 10 | p | 38821 | 38830 | *rpoC1 intron 1* | intron |
| 54 | (T)8 | 8 | p | 38940 | 38947 | *rpoC1 intron 1* | intron |
| 55 | (T)8 | 8 | p | 39136 | 39143 | *rpoC1 exon2* | cds |
| 56 | (A)9N(T)8 | 22 | ci | 40341 | 40362 | *rpoC1 exon2* | cds |
| 57 | (T)8 | 8 | p | 42856 | 42863 | *rpoC2* | cds |
| 58 | (A)11N(A)9N(A)8N(A)9 | 182 | i | 42997 | 43178 | *rpoC2* | cds |
| 59 | (A)8 | 8 | p | 44910 | 44917 | *rpoC2* | cds |
| 60 | (A)9 | 9 | p | 45238 | 45246 | *rpoC2-rps2* | spacer |
| 61 | (A)10 | 10 | p | 45508 | 45517 | *rps2* | cds |
| 62 | (A)11 | 11 | p | 46087 | 46097 | *rps2-atpI* | spacer |
| 63 | (A)12N(A)9 | 31 | i | 47258 | 47288 | *atpI-atpH* | spacer |
| 64 | (A)14N(A)8 | 28 | i | 47430 | 47457 | *atpI-atpH* | spacer |
| 65 | (A)12 | 12 | p | 47791 | 47802 | *atpI-atpH* | spacer |
| 66 | (A)8 | 8 | p | 48318 | 48325 | *atpH-atpF* | spacer |
| 67 | (T)16 | 16 | p | 48515 | 48530 | *atpH-atpF* | spacer |
| 68 | (T)8 | 8 | p | 48878 | 48885 | *atpF exon 1* | cds |
| 69 | (AATT)3N(T)8N(A)8 | 42 | ci | 48997 | 49038 | *atpF intron* | intron |
| 70 | (T)8N(A)11N(A)8 | 171 | ci | 49218 | 49388 | *atpF intron* | intron |
| 71 | (A)9 | 9 | p | 51793 | 51801 | *atpA-trnR(UCU)* | spacer |
| 72 | (T)16N(A)8 | 58 | ci | 51961 | 52018 | *trnR(UCU)-trnS(GCU)* | spacer |
| 73 | (A)8 | 8 | p | 52588 | 52595 | *trnR(UCU)-trnS(GCU)* | spacer |
| 74 | (T)9 | 9 | p | 53183 | 53191 | *trnR(UCU)-trnS(GCU)* | spacer |
| 75 | (A)10 | 10 | p | 53302 | 53311 | *trnR(UCU)-trnS(GCU)* | spacer |
| 76 | (T)10 | 10 | p | 53414 | 53423 | *trnR(UCU)-trnS(GCU)* | spacer |
| 77 | (A)8N(T)11 | 30 | ci | 53712 | 53741 | *trnS(GCU)-psbI* | spacer |
| 78 | (T)8 | 8 | p | 54181 | 54188 | *psbI-psbK* | spacer |
| 79 | (A)8 | 8 | p | 54373 | 54380 | *psbK* | cds |
| 80 | (T)10N(TA)5N(A)10 | 90 | ci | 54581 | 54670 | *psbK-trnQ(UUG)* | spacer |
| 81 | (TA)5 | 10 | p | 54827 | 54836 | *psbK-trnQ(UUG)* | spacer |
| 82 | (T)8 | 8 | p | 55098 | 55105 | *psbK-trnQ(UUG)* | spacer |
| 83 | (T)14N(A)9 | 123 | ci | 55261 | 55383 | *trnQ(UUG)-rps16* | spacer |
| 84 | (T)8 | 8 | p | 55607 | 55614 | *trnQ(UUG)-rps16* | spacer |
| 85 | (A)10 | 10 | p | 55931 | 55940 | *trnQ(UUG)-rps16* | spacer |
| 86 | (A)22 | 22 | p | 56072 | 56093 | *trnQ(UUG)-rps16* | spacer |
| 87 | (T)8 | 8 | p | 56239 | 56246 | *trnQ(UUG)-rps16* | spacer |
| 88 | (A)9N(T)8 | 64 | ci | 56661 | 56724 | *rps16-accD* | spacer |
| 89 | (T)8 | 8 | p | 57386 | 57393 | *accD* | cds |
| 90 | (A)8 | 8 | p | 57753 | 57760 | *accD* | cds |
| 91 | (T)8 | 8 | p | 58872 | 58879 | *psaI-cemA* | spacer |
| 92 | (T)8N(T)8 | 95 | ci | 58988 | 59082 | *psaI-cemA* | spacer |
| 93 | (A)8 | 8 | p | 59218 | 59225 | *psaI-cemA* | spacer |
| 94 | (A)11(T)13 | 24 | c | 59593 | 59616 | *psaI-cemA* | spacer |
| 95 | (A)8 | 8 | p | 59871 | 59878 | *psaI-cemA* | spacer |
| 96 | (T)10 | 10 | p | 60189 | 60198 | *cemA* | cds |
| 97 | (T)8 | 8 | p | 60721 | 60728 | *cemA-petA* | spacer |
| 98 | (A)9 | 9 | p | 61291 | 61299 | *petA* | cds |
| 99 | (A)8 | 8 | p | 61504 | 61511 | *petA* | cds |
| 100 | (T)13N(T)9N(AT)6N(AT)5 | 183 | ci | 62074 | 62256 | *petA-psbJ* | spacer |
| 101 | (A)9 | 9 | p | 62556 | 62564 | *petA-psbJ* | spacer |
| 102 | (ATAG)3 | 12 | p | 62986 | 62997 | *psbJ-psbL* | spacer |
| 103 | (T)8N(A)11 | 22 | ci | 64397 | 64418 | *psbE-petL* | spacer |
| 104 | (A)8 | 8 | p | 64730 | 64737 | *petL-petG* | spacer |
| 105 | (AT)7N(AT)7N(TA)6 | 49 | ci | 65119 | 65167 | *trnW(CCA)-trnP(UGG)* | spacer |
| 106 | (AT)6 | 12 | p | 65566 | 65577 | *trnP(GGG)-psaJ* | spacer |
| 107 | (T)9 | 9 | p | 66167 | 66175 | *psaJ-rpl33* | spacer |
| 108 | (TA)6 | 12 | p | 66556 | 66567 | *rpl33-rps18* | spacer |
| 109 | (A)14 | 14 | p | 66992 | 67005 | *rps18* | cds |
| 110 | (A)10N(T)12 | 44 | ci | 67126 | 67169 | *rps18-rpl20* | spacer |
| 111 | (A)10 | 10 | p | 67741 | 67750 | *rpl20-rps12* | spacer |
| 112 | (A)8N(T)10 | 83 | ci | 67890 | 67972 | *rpl20-rps12* | spacer |
| 113 | (A)8 | 8 | p | 68396 | 68403 | *rpl20-rps12* | spacer |
| 114 | (TA)6 | 12 | p | 68570 | 68581 | *rps12-clpP* | spacer |
| 115 | (T)8N(A)14N(AAT)4N(T)8N(T)8 | 213 | ci | 69220 | 69432 | *clpP intron 1* | intron |
| 116 | (T)8 | 8 | p | 70025 | 70032 | *clpP intron 2* | intron |
| 117 | (T)8 | 8 | p | 72857 | 72864 | *psbT* | cds |
| 118 | (A)8N(T)8 | 56 | ci | 73912 | 73967 | *psbH-petB* | spacer |
| 119 | (T)8N(T)10N(AAT)4 | 114 | ci | 74079 | 74192 | *psbH-petB* | spacer |
| 120 | (G)8 | 8 | p | 74505 | 74512 | *psbH-petB* | spacer |
| 121 | (T)11 | 11 | p | 75318 | 75328 | *petB-petD* | spacer |
| 122 | (T)8 | 8 | p | 75785 | 75792 | *petB-petD* | spacer |
| 123 | (T)9 | 9 | p | 75902 | 75910 | *petB-petD* | spacer |
| 124 | (AT)9 | 18 | p | 76765 | 76782 | *petD-rpoA* | spacer |
| 125 | (A)10 | 10 | p | 78390 | 78399 | *rps11-rpl36* | spacer |
| 126 | (AT)5 | 10 | p | 78593 | 78602 | *rps11-rpl37* | spacer |
| 127 | (T)11 | 11 | p | 79725 | 79735 | *rps8-rpl14* | spacer |
| 128 | (AT)5 | 10 | p | 80895 | 80904 | *rpl16-rps3* | spacer |
| 129 | (T)8N(T)9Nt(A)11 | 121 | ci | 81065 | 81185 | *rpl16-rps3* | spacer |
| 130 | (T)9 | 9 | p | 81502 | 81510 | *rpl16-rps3* | spacer |
| 131 | (TTTC)4N(T)8N(T)9N(T)11N(T)10 | 234 | ci | 81654 | 81887 | *rpl16-rps3* | spacer |
| 132 | (T)8 | 8 | p | 82426 | 82433 | *rps3* | cds |
| 133 | (T)14 | 14 | p | 82781 | 82794 | *rps3-rps19* | spacer |
| 134 | (T)9 | 9 | p | 83346 | 83354 | *rps 19* | cds |
| 135 | (A)8 | 8 | p | 89071 | 89078 | *ycf2 exon2* | cds |
| 136 | (C)8 | 8 | p | 90759 | 90766 | *ycf2 exon2* | cds |
| 137 | (A)8 | 8 | p | 91465 | 91472 | *ycf2 exon2* | cds |
| 138 | (G)8 | 8 | p | 92052 | 92059 | *ycf2 exon2* | cds |
| 139 | (A)12 | 12 | p | 92762 | 92773 | *ycf2-trnL(CAA)* | spacer |
| 140 | (AT)5 | 10 | p | 93397 | 93406 | *trnL(CAA)-ndhB* | spacer |
| 141 | (A)8 | 8 | p | 96038 | 96045 | *ndhB-rps7* | spacer |
| 142 | (T)8 | 8 | p | 96803 | 96810 | *rps7-rps12* | spacer |
| 143 | (T)9N(T)10 | 24 | ci | 98045 | 98068 | *rps12-ycf15* | spacer |
| 144 | (T)9 | 9 | p | 99103 | 99111 | *ycf15-trnV(GAC)* | spacer |
| 145 | (G)11 | 11 | p | 103199 | 103209 | *trnA(UGC)intron* | intron |
| 146 | (AG)5 | 10 | p | 107150 | 107159 | *rrn5-trnR(ACG)* | spacer |
| 147 | (A)9g(A)8 | 18 | i | 107461 | 107478 | *trnR(ACG)-trnN(GUU)* | spacer |
| 148 | (TA)5 | 10 | p | 108269 | 108278 | *trnN(GUU)-ycf1* | spacer |
| 149 | (T)9 | 9 | p | 109408 | 109416 | *ycf1* | cds |
| 150 | (A)9N(A)8 | 113 | i | 109687 | 109799 | *ycf1* | cds |
| 151 | (T)12 | 12 | p | 109921 | 109932 | *ycf1* | cds |
| 152 | (A)8 | 8 | p | 110042 | 110049 | *ycf1* | cds |
| 153 | (A)14 | 14 | p | 110332 | 110345 | *ycf1* | cds |
| 154 | (T)8 | 8 | p | 110474 | 110481 | *ycf1* | cds |
| 155 | (A)12N(A)8 | 30 | i | 110642 | 110671 | *ycf1* | cds |
| 156 | (T)8 | 8 | p | 110811 | 110818 | *ycf1* | cds |
| 157 | (A)10 | 10 | p | 111136 | 111145 | *ycf1* | cds |
| 158 | (A)7N(A)9 | 30 | i | 111280 | 111309 | *ycf1* | cds |
| 159 | (A)10N(A)8 | 52 | i | 111465 | 111516 | *ycf1* | cds |
| 160 | (A)9 | 9 | p | 111770 | 111778 | *ycf1* | cds |
| 161 | (A)8N(A)11 | 119 | i | 112207 | 112325 | *ycf1* | cds |
| 162 | (T)8N(A)11 | 82 | ci | 112707 | 112788 | *ycf1* | cds |
| 163 | (A)9 | 9 | p | 113405 | 113413 | *ycf1* | cds |
| 164 | (A)12 | 12 | p | 113599 | 113610 | *ycf1* | cds |
| 165 | (T)14N(A)14(AATA)2N(A)10N(AT)6N(A)8 | 298 | ci | 113920 | 114217 | *ycf1-rps15* | spacer |
| 166 | (T)9 | 9 | p | 115761 | 115769 | *orf188* | cds |
| 167 | (T)10 | 10 | p | 116670 | 116679 | *orf188-ndhA* | cds |
| 168 | (A)9gtgatcccccc(T)8 | 28 | ci | 117036 | 117063 | *orf188-ndhA* | cds |
| 169 | (TTTA)3 | 12 | p | 117269 | 117280 | *orf188-ndhA* | cds |
| 170 | (A)8N(TA)6 | 61 | ci | 118654 | 118714 | *ndhI-ndhG* | spacer |
| 171 | (T)8 | 8 | p | 119579 | 119586 | *ndhG-ndhE* | spacer |
| 172 | (A)8 | 8 | p | 120008 | 120015 | *ndhE-psaC* | spacer |
| 173 | (A)9N(T)8 | 24 | ci | 120601 | 120624 | *psaC-ndhD* | spacer |
| 174 | (A)8 | 8 | p | 121568 | 121575 | *ndhD* | cds |
| 175 | (A)10N(A)9 | 40 | i | 122190 | 122229 | *ndhD-ccsA* | spacer |
| 176 | (A)8 | 8 | p | 122739 | 122746 | *ccsA* | cds |
| 177 | (A)8 | 8 | p | 123213 | 123220 | *ccsA* | cds |
| 178 | (T)8 | 8 | p | 123948 | 123955 | *rpl32* | cds |
| 179 | (T)9 | 9 | p | 124109 | 124117 | *rpl32-ndhF* | spacer |
| 180 | (T)8 | 8 | p | 125077 | 125084 | *ndhF* | cds |
| 181 | (A)9 | 9 | p | 125810 | 125818 | *ndhF* | cds |
| 182 | (AT)5 | 10 | p | 127230 | 127239 | *ycf1-trnN(GUU)* | spacer |
| 183 | (T)8N(T)9 | 18 | i | 128031 | 128048 | *trnN(GUU)-trnR(ACG)* | spacer |
| 184 | (CT)5 | 10 | p | 128350 | 128359 | *trnR(ACG)-rrn5* | spacer |
| 185 | (C)11 | 11 | p | 132300 | 132310 | *trnA(UGC)intron* | intron |
| 186 | (A)9 | 9 | p | 136398 | 136406 | *trnV(GAC)-ycf15* | spacer |
| 187 | (A)9 | 9 | p | 137456 | 137464 | *ycf15-rps12* | spacer |
| 188 | (A)8 | 8 | p | 138699 | 138706 | *rps12-rps7* | spacer |
| 189 | (T)8 | 8 | p | 139464 | 139471 | *rps7-ndhB* | spacer |
| 190 | (AT)5 | 10 | p | 142103 | 142112 | *ndhB-trnL(CAA)* | spacer |
| 191 | (T)16 | 16 | p | 142736 | 142751 | *trnL(CAA)-ycf2* | spacer |
| 192 | (C)8 | 8 | p | 143450 | 143457 | *ycf2 exon2* | cds |
| 193 | (T)8 | 8 | p | 144037 | 144044 | *ycf2 exon2* | cds |
| 194 | (G)8 | 8 | p | 144743 | 144750 | *ycf2 exon2* | cds |
| 195 | (T)8 | 8 | p | 146431 | 146438 | *ycf2 exon2* | cds |
| 196 | (A)9 | 9 | p | 152155 | 152163 | *rpl2-trnH(GUG)* | spacer |

p- perfect repeat, i- imperfect repeat, c- compound repeat, ci- compound imperfect repeat.
