# Supplementary material for: Chloroplast Genome Sequence of Pigeonpea (Cajanus cajan (L.) Millspaugh) and Cajanus scarabaeoides (L.) Thouars: Genome Organization and Comparison with Other Legumes
Source: Front Plant Sci. 2016 Dec 9;7:1847. doi: 10.3389/fpls.2016.01847 (PMC5145887; doi:10.3389/fpls.2016.01847)
Supplement: Supplementary file 9 [file Table9.DOCX]

**Supplementary Table S9- SSRs found in *Cajanus cajan***

| **SSR No.** | **SSR motif** | **size** | **SSR type** | **start** | **End** | ***Location*** | **Genomic region** |
| --- | --- | --- | --- | --- | --- | --- | --- |
| 1 | (T)8 | 8 | p | 1587 | 1594 | *psbA-trnK(UUU)* | spacer |
| 2 | (A)8 | 8 | p | 1933 | 1940 | *trnK(UUU) intron* | intron |
| 3 | (T)9 | 9 | p | 2284 | 2292 | *trnK(UUU) intron* | intron |
| 4 | (A)8 | 8 | p | 2826 | 2833 | *trnK(UUU) intron* | intron |
| 5 | (A)8 | 8 | p | 2951 | 2958 | *trnK(UUU) intron* | intron |
| 6 | (A)8 | 8 | p | 3477 | 3484 | *trnK(UUU) intron* | intron |
| 7 | (T)10N(A)8 | 44 | c | 3751 | 3794 | *trnK(UUU) intron* | intron |
| 8 | (A)13 | 13 | p | 3973 | 3985 | *trnK(UUU) intron* | intron |
| 9 | (A)8N(A)8 | 83 | c | 4352 | 4434 | *trnK(UUU)-rbcL* | spacer |
| 10 | (TATTA)3 | 15 | p | 4549 | 4563 | *trnK(UUU)-rbcL* | spacer |
| 11 | (A)8N(AT)5N(AAAT)3 | 51 | ci | 5016 | 5066 | *trnK(UUU)-rbcL* | spacer |
| 12 | (AT)18 | 36 | p | 5234 | 5269 | *trnK(UUU)-rbcL* | spacer |
| 13 | (T)12N(A)9N(A)8N(A)10 | 187 | ci | 7082 | 7268 | *rbcL-atpB* | spacer |
| 14 | (A)10 | 10 | p | 7423 | 7432 | *rbcL-atpB* | spacer |
| 15 | (T)9N(T)10N(A)10 | 108 | ci | 7620 | 7727 | *atpB* | cds |
| 16 | (T)10 | 10 | p | 10008 | 10017 | *trnV(UAC) intron* | intron |
| 17 | (A)11 | 11 | p | 11244 | 11254 | *ndhK* | cds |
| 18 | (T)8N(A)9 | 61 | ci | 11935 | 11995 | *ndhK-ndhJ* | spacer |
| 19 | (TA)5N(A)8 | 32 | ci | 13068 | 13099 | *ndhJ-trnF(GAA)* | spacer |
| 20 | (T)8N(A)8 | 24 | ci | 13457 | 13480 | *trnF(GAA)-trnL(UAA)* | spacer |
| 21 | (T)11 | 11 | p | 14456 | 14466 | *trnL(UAA)-trnT(UGU)* | spacer |
| 22 | (AT)5N(TA)6 | 121 | ci | 14703 | 14823 | *trnL(UAA)-trnT(UGU)* | spacer |
| 23 | (A)9N(T)8 | 73 | ci | 15187 | 15259 | *trnT(UGU)-rps4* | spacer |
| 24 | (TA)6N(A)9N(A)9 | 81 | ci | 16755 | 16835 | *ycf3 intron1* | intron |
| 25 | (A)11 | 11 | p | 17769 | 17779 | *ycf3 intron2* | intron |
| 26 | (AT)5 | 10 | p | 18162 | 18171 | *ycf3 intron2* | intron |
| 27 | (T)8N(A)8N(A)9 | 42 | ci | 18334 | 18375 | *ycf3 intron2* | intron |
| 28 | (A)9 | 9 | p | 18833 | 18841 | *ycf3-psaA* | spacer |
| 29 | (A)8 | 8 | p | 18955 | 18962 | *ycf3-psaA* | spacer |
| 30 | (T)8 | 8 | p | 24219 | 24226 | *rps14-trnfM(CAU)* | spacer |
| 31 | (T)8 | 8 | p | 24489 | 24496 | *trnG(UCC)-IhbA* | spacer |
| 32 | (T)9N(TAT)5 | 55 | ci | 24602 | 24656 | *trnG(UCC)-IhbA* | spacer |
| 33 | (TA)8 | 16 | p | 24832 | 24847 | *trnG(UCC)-IhbA* | spacer |
| 34 | (T)8 | 8 | p | 25388 | 25395 | *IhbA-trnS(UGA)* | spacer |
| 35 | (A)13 | 13 | p | 25766 | 25778 | *trnS(UGA)-psbC* | spacer |
| 36 | (A)8 | 8 | p | 28470 | 28477 | *psbD-trnT(GGU)* | spacer |
| 37 | (T)8N(T)10 | 21 | i | 28596 | 28616 | *psbD-trnT(GGU)* | spacer |
| 38 | (TTA)5N(ATT)5N(A)8 | 73 | ci | 28792 | 28864 | *psbD-trnT(GGU)* | spacer |
| 39 | (A)8 | 8 | p | 29252 | 29259 | *psbD-trnT(GGU)* | spacer |
| 40 | (T)9N(T)8N(A)9 | 164 | ci | 29387 | 29550 | *psbD-trnT(GGU)* | spacer |
| 41 | (T)15N(T)8 | 118 | i | 30195 | 30312 | *trnM(CAU)-trnE(UUC)* | spacer |
| 42 | (T)8 | 8 | p | 30726 | 30733 | *trnY(GUA)-trnD(GUC)* | spacer |
| 43 | (A)8N(A)8N(T)9 | 149 | ci | 31721 | 31869 | *psbM-petN* | spacer |
| 44 | (A)8 | 8 | p | 31987 | 31994 | *psbM-petN* | spacer |
| 45 | (T)9N(A)10 | 92 | ci | 32565 | 32656 | *petN-trnC(GCA)* | spacer |
| 46 | (T)8N(T)8 | 40 | i | 32786 | 32825 | *petN-trnC(GCA)* | spacer |
| 47 | (T)8 | 8 | p | 32988 | 32995 | *petN-trnC(GCA)* | spacer |
| 48 | (T)9N(A)9 | 99 | ci | 33437 | 33535 | *petN-trnC(GCA)* | spacer |
| 49 | (T)8N(A)9N(T)8N(A)9 | 215 | ci | 33679 | 33893 | *trnC(GCA)-rpoB* | spacer |
| 50 | (AT)7N(T)10 | 62 | ci | 33995 | 34056 | *trnC(GCA)-rpoB* | spacer |
| 51 | (G)8 | 8 | p | 35029 | 35036 | *rpoB* | cds |
| 52 | (A)10N(A)10 | 114 | i | 35205 | 35318 | *rpoB* | cds |
| 53 | (A)8 | 8 | p | 37879 | 37886 | *rpoC1 exon1* | cds |
| 54 | (A)13 | 13 | p | 38468 | 38480 | *rpoC1 intron* | intron |
| 55 | (A)10 | 10 | p | 38829 | 38838 | *rpoC1 intron* | intron |
| 56 | (T)8 | 8 | p | 38948 | 38955 | *rpoC1 intron* | intron |
| 57 | (T)8 | 8 | p | 39144 | 39151 | *rpoC1 exon2* | cds |
| 58 | (A)9N(T)8 | 22 | ci | 40349 | 40370 | *rpoC1exon2* | cds |
| 59 | (T)8 | 8 | p | 42864 | 42871 | *rpoC2* | cds |
| 60 | (A)11N(A)9N(A)8N(A)9 | 182 | ci | 43005 | 43186 | *rpoC2* | cds |
| 61 | (A)9 | 9 | p | 44918 | 44926 | *rpoC2* | cds |
| 62 | (A)9 | 9 | p | 45246 | 45254 | *rpoC2-rps2* | spacer |
| 63 | (A)10 | 10 | p | 45516 | 45525 | *rps2* | cds |
| 64 | (A)11 | 11 | p | 46095 | 46105 | *rps2-atpI* | spacer |
| 65 | (A)12N(A)8 | 30 | i | 47268 | 47297 | *atpI-atpH* | spacer |
| 66 | (A)15 | 15 | p | 47438 | 47452 | *atpI-atpH* | spacer |
| 67 | (A)20 | 20 | p | 47793 | 47812 | *atpI-atpH* | spacer |
| 68 | (A)8 | 8 | p | 48328 | 48335 | *atpH-atpF* | spacer |
| 69 | (T)16 | 16 | p | 48525 | 48540 | *atpH-atpF* | spacer |
| 70 | (T)8 | 8 | p | 48894 | 48901 | *atpF exon1* | cds |
| 71 | (AATT)3N(T)8N(A)8 | 41 | ci | 49013 | 49053 | *atpF intron* | intron |
| 72 | (T)8N(A)11N(A)8 | 171 | ci | 49233 | 49403 | *atpF intron* | intron |
| 73 | (A)8 | 8 | p | 49528 | 49535 | *atpF intron* | intron |
| 74 | (A)8N(A)18N(A)8 | 43 | i | 51795 | 51837 | *atpA-trnR(UCU)* | spacer |
| 75 | (T)16N(A)8 | 58 | ci | 51976 | 52033 | *trnR(UCU)-trnS(GCU)* | spacer |
| 76 | (A)8 | 8 | p | 52603 | 52610 | *trnR(UCU)-trnS(GCU)* | spacer |
| 77 | (T)8 | 8 | p | 53198 | 53205 | *trnR(UCU)-trnS(GCU)* | spacer |
| 78 | (T)10 | 10 | p | 53429 | 53438 | *trnR(UCU)-trnS(GCU)* | spacer |
| 79 | (A)8N(T)11 | 30 | ci | 53727 | 53756 | *trnS(GCU)-psbI* | spacer |
| 80 | (A)8 | 8 | p | 54385 | 54392 | *psbK* | cds |
| 81 | (T)10N(TA)5N(A)11 | 93 | ci | 54593 | 54685 | *psbK-trnQ(UUG)* | spacer |
| 82 | (TA)5 | 10 | p | 54842 | 54851 | *psbK-trnQ(UUG)* | spacer |
| 83 | (T)8 | 8 | p | 55113 | 55120 | *trnQ(UUG)-rps16* | spacer |
| 84 | (T)14N(A)9 | 123 | ci | 55276 | 55398 | *trnQ(UUG)-rps16* | spacer |
| 85 | (T)8 | 8 | p | 55622 | 55629 | *trnQ(UUG)-rps16* | spacer |
| 86 | (A)10 | 10 | p | 55946 | 55955 | *trnQ(UUG)-rps16* | spacer |
| 87 | (A)22 | 22 | p | 56087 | 56108 | *trnQ(UUG)-rps16* | spacer |
| 88 | (T)8 | 8 | p | 56254 | 56261 | *trnQ(UUG)-rps16* | spacer |
| 89 | (A)9N(T)9 | 65 | ci | 56676 | 56740 | *rps16-accD* | spacer |
| 90 | (T)8 | 8 | p | 57402 | 57409 | *accD exon2* | cds |
| 91 | (A)8 | 8 | p | 57769 | 57776 | *accD exon2* | cds |
| 92 | (A)10 | 10 | p | 58692 | 58701 | *accD-psaI* | spacer |
| 93 | (T)8N(T)8 | 95 | i | 59005 | 59099 | *ycf4* | cds |
| 94 | (A)8 | 8 | p | 59235 | 59242 | *ycf4-cemA* | spacer |
| 95 | (A)11cc(T)11 | 24 | ci | 59610 | 59633 | *ycf4-cemA* | spacer |
| 96 | (A)8 | 8 | p | 59888 | 59895 | *ycf4-cemA* | spacer |
| 97 | (T)10 | 10 | p | 60206 | 60215 | *cemA* | cds |
| 98 | (T)8N(T)9 | 29 | i | 60738 | 60766 | *cemA-petA* | spacer |
| 99 | (A)9 | 9 | p | 61307 | 61315 | *petA* | cds |
| 100 | (A)8 | 8 | p | 61520 | 61527 | *petA* | cds |
| 101 | (T)9N(AT)6N(AT)5 | 135 | ci | 62139 | 62273 | *petA-psbJ* | spacer |
| 102 | (T)8 | 8 | p | 62442 | 62449 | *petA-psbJ* | spacer |
| 103 | (A)9N(A)8 | 89 | i | 62578 | 62666 | *petA-psbJ* | spacer |
| 104 | (ATAG)3 | 12 | p | 62009 | 62020 | *psbJ-psbL* | spacer |
| 105 | (T)9 | 9 | p | 64419 | 64427 | *psbE-petL* | spacer |
| 106 | (A)8 | 8 | p | 64752 | 64759 | *petL-petG* | spacer |
| 107 | (AT)6N(TA)6 | 47 | ci | 65141 | 65187 | *trnW(CCA)-trnP(UGG)* | spacer |
| 108 | (AT)6 | 12 | p | 65586 | 65597 | *trnP(GGG)-psaJ* | spacer |
| 109 | (T)9 | 9 | p | 66187 | 66195 | *psaJ-rpl33* | spacer |
| 110 | (TA)6 | 12 | p | 66576 | 66587 | *rpl33-rps18* | spacer |
| 111 | (A)14 | 14 | p | 67012 | 67025 | *rps18* | cds |
| 112 | (A)10N(T)13 | 46 | ci | 67146 | 67191 | *rps18-rpl20* | spacer |
| 113 | (A)10 | 10 | p | 67763 | 67772 | *rpl20-rps12* | spacer |
| 114 | (A)8N(T)10 | 83 | ci | 67912 | 67994 | *rpl20-rps13* | spacer |
| 115 | (A)8 | 8 | p | 68418 | 68425 | *rpl20-rps14* | spacer |
| 116 | (TA)6 | 12 | p | 68592 | 68603 | *rps12-clpP* | spacer |
| 117 | (T)8N(A)14N(AAT)4N(T)8N(T)8 | 213 | ci | 69242 | 69454 | *clpP intron1* | intron |
| 118 | (T)8 | 8 | p | 70047 | 70054 | *clpP intron2* | intron |
| 119 | (T)8 | 8 | p | 72879 | 72886 | *psbB-psbT* | spacer |
| 120 | (A)8N(T)10 | 58 | ci | 73934 | 73991 | *psbH-petB* | spacer |
| 121 | (T)8N(T)10N(AAT)4 | 114 | ci | 74103 | 74216 | *psbH-petB* | spacer |
| 122 | (G)8 | 8 | p | 74529 | 74536 | *psbH-petB* | spacer |
| 123 | (T)11 | 11 | p | 75342 | 75352 | *petB-petD* | spacer |
| 124 | (T)8 | 8 | p | 75809 | 75816 | *petB-petD* | spacer |
| 125 | (T)13 | 13 | p | 75926 | 75938 | *petB-petD* | spacer |
| 126 | (AT)9 | 18 | p | 76789 | 76806 | *petD-rpoA* | spacer |
| 127 | (A)11 | 11 | p | 78413 | 78423 | *rps11-rpl36* | spacer |
| 128 | (AT)5(ATA)5* | 23 | c | 78618 | 78640 | *rps11-rpl37* | spacer |
| 129 | (T)9 | 9 | p | 79752 | 79760 | *rps8-rpl14* | spacer |
| 130 | (AT)5 | 10 | p | 80920 | 80929 | *rpl16-rps3* | spacer |
| 131 | (T)8N(T)9N(A)11 | 121 | ci | 81090 | 81210 | *rpl16-rps3* | spacer |
| 132 | (T)9 | 9 | p | 81527 | 81535 | *rpl16-rps3* | spacer |
| 133 | (TTTC)4N(T)8N(T)9N(T)12N(T)9 | 235 | ci | 81679 | 81913 | *rpl16-rps3* | spacer |
| 134 | (T)8 | 8 | p | 82454 | 82461 | *rps3* | cds |
| 135 | (T)12 | 12 | p | 82809 | 82820 | *rps3-rps19* | spacer |
| 136 | (T)9 | 9 | p | 83378 | 83386 | *rps19* | cds |
| 137 | (A)8 | 8 | p | 89103 | 89110 | *ycf2 exon2* | cds |
| 138 | (C)8 | 8 | p | 90791 | 90798 | *ycf2 exon2* | cds |
| 139 | (A)8 | 8 | p | 91497 | 91504 | *ycf2 exon2* | cds |
| 140 | (G)8 | 8 | p | 92084 | 92091 | *ycf2 exon2* | cds |
| 141 | (A)13 | 13 | p | 92793 | 92805 | *ycf2-trnL(CAA)* | spacer |
| 142 | (AT)5 | 10 | p | 93429 | 93438 | *ndhB exon1* | cds |
| 143 | (A)8 | 8 | p | 96068 | 96075 | *ndhB-rps7* | spacer |
| 144 | (T)8 | 8 | p | 96833 | 96840 | *rps7-rps12* | spacer |
| 145 | (T)9 | 9 | p | 98075 | 98083 | *rps12-ycf15* | spacer |
| 146 | (T)10 | 10 | p | 99134 | 99143 | *ycf15-trnV(GAC)* | spacer |
| 147 | (G)10 | 10 | p | 103231 | 103240 | *trnA(UGC) intron* | intron |
| 148 | (AG)5 | 10 | p | 107181 | 107190 | *rrn5-trnR(ACG)* | spacer |
| 149 | (A)10N(A)10 | 21 | ci | 107492 | 107512 | *rrn5-trnR(ACG)* | spacer |
| 150 | (TA)5 | 10 | p | 108301 | 108310 | *trnN(GUU)-ycf1* | spacer |
| 151 | (T)9 | 9 | p | 109440 | 109448 | *ycf1* | cds |
| 152 | (A)9N(A)8 | 113 | ci | 109719 | 109831 | *ycf1* | cds |
| 153 | (T)12 | 12 | p | 109953 | 109964 | *ycf1* | cds |
| 154 | (A)8 | 8 | p | 110074 | 110081 | *ycf1* | cds |
| 155 | (A)14 | 14 | p | 110364 | 110377 | *ycf1* | cds |
| 156 | (T)8 | 8 | p | 110506 | 110513 | *ycf1* | cds |
| 157 | (A)12N(A)8 | 30 | ci | 110674 | 110703 | *ycf1* | cds |
| 158 | (T)8 | 8 | p | 110843 | 110850 | *ycf1* | cds |
| 159 | (A)10 | 10 | p | 111168 | 111177 | *ycf1* | cds |
| 160 | (A)9N(A)8 | 52 | ci | 111333 | 111384 | *ycf1* | cds |
| 161 | (A)10N(A)8 | 52 | ci | 111497 | 111548 | *ycf1-rps15* | spacer |
| 162 | (A)9 | 9 | p | 111802 | 111810 | *ycf1-rps15* | spacer |
| 163 | (A)8N(A)12 | 120 | ci | 112240 | 112359 | *ycf1-rps15* | spacer |
| 164 | (T)8Ng(A)11 | 83 | ci | 112741 | 112823 | *ycf1-rps15* | spacer |
| 165 | (A)10 | 10 | p | 113440 | 113449 | *ycf1-rps15* | spacer |
| 166 | (A)9 | 9 | p | 113635 | 113643 | *ycf1-rps15* | spacer |
| 167 | (T)13N(A)14(AATA)3N(A)10N(AT)6N(A)8 | 298 | ci | 113958 | 114255 | *ycf1-rps15* | spacer |
| 168 | (T)9 | 9 | p | 115799 | 115807 | *orf88* | cds |
| 169 | (T)10 | 10 | p | 116708 | 116717 | *orf-ndhA* | cds |
| 170 | (A)9N(T)8 | 28 | ci | 117074 | 117101 | *orf-ndhA* | cds |
| 171 | (T)9N(TTTA)3 | 58 | ci | 117262 | 117319 | *orf-ndhA* | cds |
| 172 | (A)8N(TA)6 | 61 | ci | 118694 | 118754 | *ndhI-ndhG* | spacer |
| 173 | (T)8 | 8 | p | 119619 | 119626 | *ndhG-ndhE* | spacer |
| 174 | (A)8 | 8 | p | 120048 | 12055 | *ndhE-psaC* | spacer |
| 175 | (A)9N(T)8 | 24 | ci | 120641 | 120664 | *psaC-ndhD* | spacer |
| 176 | (A)8 | 8 | p | 121608 | 121615 | *ndhD* | cds |
| 177 | (A)9 | 9 | p | 122261 | 122269 | *ndhD-ccsA* | spacer |
| 178 | (A)8 | 8 | p | 122779 | 122786 | *ccsA* | cds |
| 179 | (A)8 | 8 | p | 123253 | 123260 | *ccsA* | cds |
| 180 | (T)8 | 8 | p | 123988 | 123995 | *rpl32* | cds |
| 181 | (T)9N(A)8 | 87 | ci | 124149 | 124235 | *rpl32-ndhF* | spacer |
| 182 | (T)8 | 8 | p | 125118 | 125125 | *ndhF* | cds |
| 183 | (A)9 | 9 | p | 125851 | 125855 | *ndhF* | cds |
| 184 | (AT)5 | 10 | p | 127271 | 127280 | *ycf1-trnN(GUU)* | spacer |
| 185 | (T)8c(T)10 | 19 | ci | 128072 | 128090 | *trnN(GUU)-trnR(ACG)* | spacer |
| 186 | (CT)5 | 10 | p | 128392 | 128401 | *trnR(ACG)-rrn5* | spacer |
| 187 | (C)10 | 10 | p | 132342 | 132351 | *trnA(UGC)intron* | intron |
| 188 | (A)10 | 10 | p | 136439 | 136448 | *trnV(GAC)-ycf15* | spacer |
| 189 | (A)12 | 12 | p | 137499 | 137510 | *ycf15-rps12* | spacer |
| 190 | (A)8 | 8 | p | 138742 | 138749 | *rps12-rps7* | spacer |
| 191 | (T)8 | 8 | p | 139507 | 139514 | *rps7-ndhB* | spacer |
| 192 | (AT)5 | 10 | p | 142144 | 142153 | *ndhB-trnL(CAA)* | spacer |
| 193 | (T)16 | 16 | p | 142777 | 142792 | *trnL(CAA)-ycf2* | spacer |
| 194 | (C)8 | 8 | p | 143491 | 143498 | *ycf2 exon2* | cds |
| 195 | (T)8 | 8 | p | 144078 | 144085 | *ycf2 exon2* | cds |
| 196 | (G)8 | 8 | p | 144784 | 144791 | *ycf2 exon2* | cds |
| 197 | (T)8 | 8 | p | 146472 | 146479 | *ycf2 exon2* | cds |
| 198 | (A)9 | 9 | p | 152196 | 152204 | *rpl2-trnH(GUG)* | spacer |

p- perfect repeat, i- imperfect repeat, c- compound repeat, ci- coumpound imperfect repeat.
